# A PCR-Based Technique to Track the Geographic Origin of *Plasmodium falciparum* With 23-SNP Barcode Analysis

**DOI:** 10.3389/fpubh.2021.649170

**Published:** 2021-04-16

**Authors:** Fengyue Hu, Qicheng Zou, Yinyue Li, Guoding Zhu, Huayun Zhou, Meihua Zhang, Fang Tian, Yaobao Liu, Feng Lu

**Affiliations:** ^1^Jiangsu Key Laboratory of Experimental and Translational Non-coding RNA Research, School of Medicine, Yangzhou University, Yangzhou, China; ^2^National Health Commission Key Laboratory of Parasitic Disease Control and Prevention, Jiangsu Institute of Parasitic Diseases, Wuxi, China; ^3^Jiangsu Provincial Key Laboratory on Parasite and Vector Control Technology, Jiangsu Institute of Parasitic Diseases, Wuxi, China; ^4^Department of Clinical Laboratory, Affiliated Hospital of Yangzhou University, Yangzhou, China; ^5^Jiangsu Key Laboratory of Zoonosis, Jiangsu Co-Innovation Center for Prevention and Control of Important Animal Infectious Diseases and Zoonoses, Yangzhou, China

**Keywords:** *Plasmodium falciparum*, mitochondrion, apicoplast, SNP, barcode

## Abstract

Increased population movement has increased the risk of reintroducing parasites to elimination areas and also dispersing drug-resistant parasites to new regions. Therefore, reliable and repeatable methods to trace back to the source of imported infections are essential. The recently developed 23-single-nucleotide polymorphism (SNP) barcode from organellar genomes of mitochondrion (*mt*) and apicoplast (*apico*) provides a valuable tool to locate the geographic origin of *Plasmodium falciparum*. This study aims to explore the feasibility of using the 23-SNP barcode for tracking *P. falciparum* by polymerase chain reaction and sequencing, while providing geographical haplotypes of isolates that originated from Central Africa. Based on 23-SNP barcode analysis, SNPs were found at seven loci; 27 isolates were confirmed to have originated in West Africa, and this study also showed four isolates from Central Africa (Equatorial Guinea, 3; Republic of Congo, 1) that originated in East Africa. This study provides the sequence data from Central Africa and fills 23-SNP barcode data gaps of sample origins.

## Introduction

Malaria is a serious public health problem in tropical and subtropical areas, with an estimated 228 million cases of malaria occurring worldwide [(1), available online at: https://www.who.int/publications/i/item/9789241565721]. Among the five species of *Plasmodium* that infect humans, *Plasmodium falciparum* is the most dangerous one, causing high levels of mortality and morbidity worldwide, particularly in sub-Saharan Africa. With today's ease of transmissibility, the emergence and spread of artemisinin-resistant *P. falciparum* threatens malaria eradication (2–4). Increased population movement has increased the risk of reintroducing parasites to elimination areas and dispersing drug-resistant parasites to new regions. To facilitate a better response for this new challenge and to understand it well, except drug sensitivity monitoring, the geographic origins of imported malaria need be tracked accurately and in good time. Reliable and repeatable methods to trace back to the source of imported infections are therefore essential.

In the past decade, the single-nucleotide polymorphism (SNP) barcode has been developed as a forceful genotyping technique to investigate the origin of *Plasmodium* (5). The first *P. falciparum* molecular barcode was composed of 24 SNPs, which in combination created a unique and concise signature to differentiate recrudescence from reinfection from malaria parasites or to monitor distribution and frequency of specific parasites in patients (6). However, these nuclear SNPs are constrained by a lack of geographic specificity and frequent recombination. The establishment of a 23-SNP barcode using polymorphisms from mitochondrion (*mt*) and apicoplast (*apico*) genomes provides evidence that they are non-recombining and coinherited and are capable of identifying the geographic origin of *P. falciparum* strains (7). The 23-SNP barcoding strategy was 92% accurate in identifying the continental origin of *P. falciparum* samples from West and East Africa, Southeast Asia, Oceania, and South America (7). However, feasible and conventional methods for the 23-SNP barcode are lacking, and information about the haplotypes of isolates from Central Africa is rare. To conquer such a plight, we designed a simple 23-SNP barcode based on polymerase chain reaction (PCR) and sequencing, and assayed the samples from *P. falciparum* malaria cases imported from sub-Saharan Africa.

## Materials and Methods

### Study Sites and Participants

**Figure 1:**
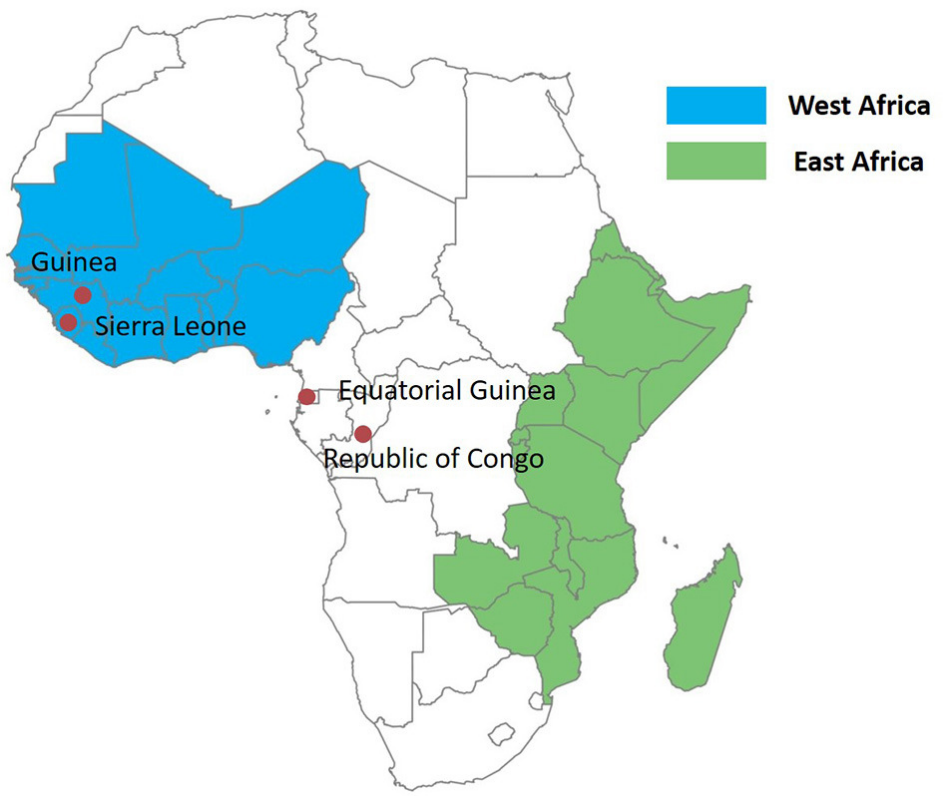
Map of Africa showing the countries where *Plasmodium falciparum* isolates were imported from: Equatorial Guinea (*n* = 29), Guinea (*n* = 1), the Republic of Congo (*n* = 1), Sierra Leone (*n* = 1).

The study was carried out in Jiangsu Province, China, where only imported malaria cases have been reported since 2013. The study was approved by the institutional review board of Jiangsu Institute of Parasitic Diseases (IRB00004221), Wuxi, China. Samples were collected from imported malaria cases. Imported cases were identified based on the travel history of the patients (travel to a malaria-endemic country within the previous month of illness onset); the last country visited with ongoing malaria transmission was taken as the potential location of infection (8). All of the malaria cases were routinely confirmed by microscopy and PCR through the malaria diagnosis reference laboratory in Jiangsu Institute of Parasitic Diseases. The 32 samples included in this study are a subset of the 765 imported malaria samples analyzed earlier (3); 21 mutated isolates were selected for this study. In addition, 11 isolates with wild-type *Pf kelch13* from Equatorial Guinea were also random selected for this barcode assay. Together, 32 samples, including 30 samples from Central Africa (29 samples from Equatorial Guinea, 1 from Guinea), and 2 samples from West Africa (Republic of Congo, Sierra Leone) as reference, were enrolled in this study (Figure 1). Figure 1 was produced using Microsoft Excel and SPSS software 26.0 for Windows.

### PCR and Sequencing of the Targets

Genomic DNA of *P. falciparum* isolates was extracted from the whole blood samples on the QIAcube Connect platform, using a QIAamp DNA Blood Kit (Qiagen, Valencia, CA) according to the manufacturer's instructions. From the 200 μL of input whole blood sample, a final 100 μL of elution volume was obtained. The gDNA was used for PCR amplification with gene-specific primers to amplify SNP loci targets within the *mt* and *apico P. falciparum* genomes. Primers were designed manually on the basis of geographically informative barcoding; the reference sequences used in this study were from the database of GenBank (https://www.ncbi.nlm.nih.gov/nucleotide/); AY282930 (5,949 bp) was selected as the reference sequence for *mt*, and X95276 (1–14,009 bp) combined the reverse sequence of X95275 (14,010–29,430 bp) for *apico* (9) (Appendix 1 in Supplementary Material).

**Figure 2:**
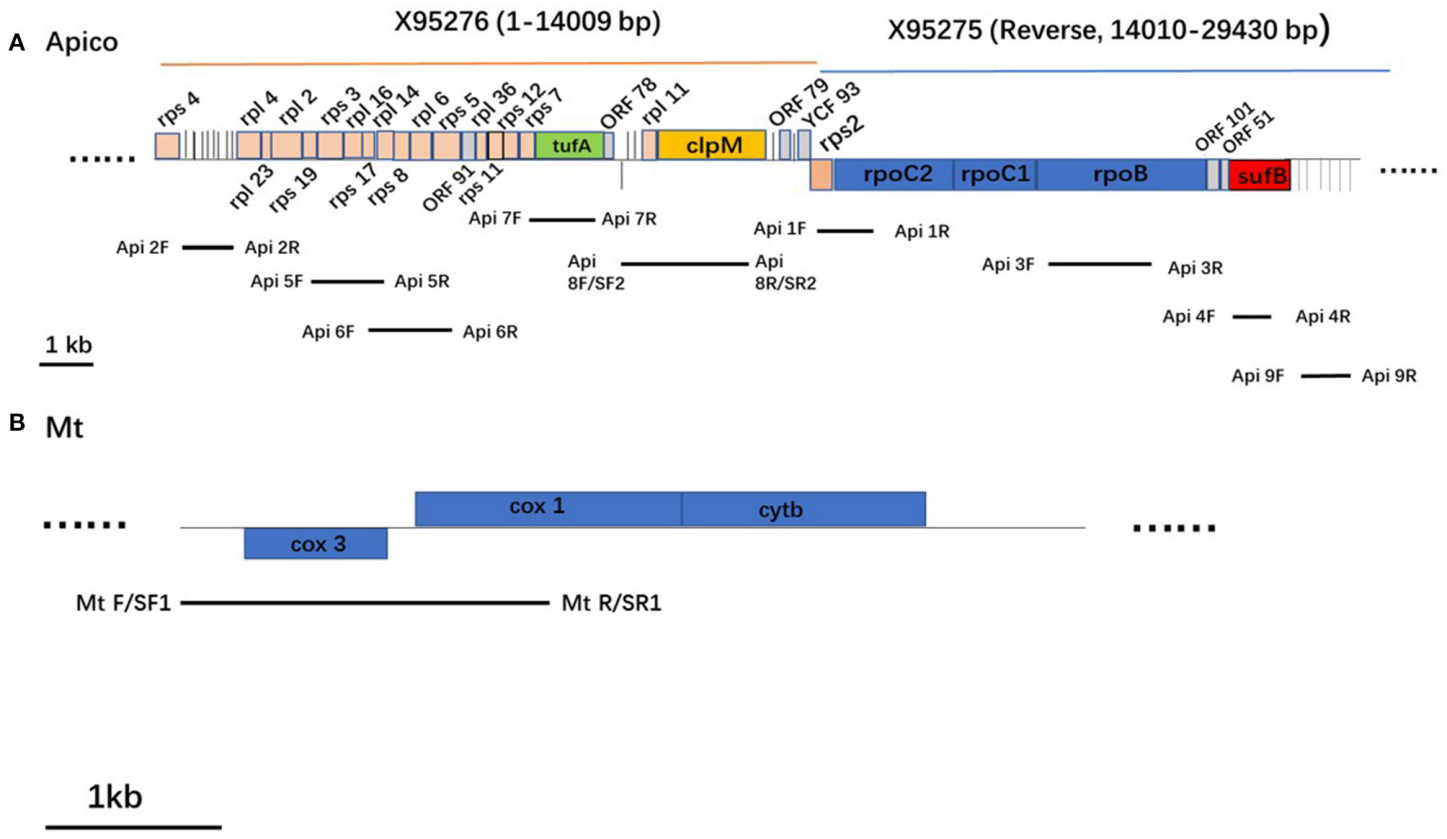
Schematic representation of the apicoplast genome **(A)** and mitochondrion genome **(B)** in *Plasmodium falciparum*. Protein coding genes are boxed and color-coded. tRNAs are represented by vertical lines. Genes shown above the horizontal line in each genome are transcribed left to right, and those below are transcribed from right to left.

A total of 10 sets of primers that covered the sites of the 23-SNP barcode were included in this study; detailed primer sequences are shown in Table 1, and the target sequences are shown (Figure 2 and Appendix 2 in Supplementary Material). Amplifications were performed in a reaction mixture that contained 25 μL of 2 × PCR buffer for KOD FX, 10 μL of 2 mM dNTPs, 1.5 μL each of 10 μM Primer-F and Primer-R, 3 μL of template DNA, 1 μL of 1.0 U/μL KOD FX, and 8 μL of double-distilled water. The PCR was performed with initial denature at 98°C for 2 min, and 35 cycles of 98°C for 10 s, 58°C for 30 s, and 68°C for 2 min and ending with a final extension of 5 min at 68°C. The PCR products were purified and sequenced. Double-strand capillary sequencing of PCR products was performed on an Applied Biosystems 3,730 sequence analyzer with the sequencing primers (Table 1). The deduced amino acid sequences were aligned and analyzed with the Lasergene® software (DNASTAR, Madison, WI).

**Table 1:** Primers used for polymerase chain reaction (PCR) amplification and sequencing of *mt/apico*.

	**Primer sequence (5^**′**^ → 3^**′**^)**	**Amplified position**	**Amplicon**
*mt*	F: AAGCTTTTGGTATCTCGTAATGTAGAA R^*^: TTAGCAATAACATTCCTGATGTAATGA SF1: CCTTCTCGCCATTTGATAGC SR1: GGCGAACCTTCTTACCGTTAT	*mt*772, *mt*853, *mt*973, *mt*1283, *mt*2383	1–2,650
*apico*	F: TTAGTTAATAATCCAGAAACCCATTT	*apico*15131	14,392–15,247
1	R^*^: GTGGATATTCTTTACATACAGA		
	F: TGCCTGAGTGGTTAAAAGGAA	*apico*2122	1,473–2,278
2	R^*^: Ccctgtttacatacaggtg		
	F: TCTTTGACCCCCTTGTTTTG	*apico*20831, *apico*21188	19,599–21,277
3	R^*^: ctaatgaaggattaacttgtgg		
	F^*^: Gaagctgtacatccttct	*apico*23803	23,595–24,104
4	R: Tatatttaaattacctgattggaa		
	F^*^: AAGTACATCCTTTAATATTTAGAGG	*apico*4370, *apico*4878, *apico*4945, *apico*5005	3,857–5,257
5	R^*^: CCTATAACTATATCACCATATTTAGG		
	F^*^: GTAGGTACTAAAAGTAATAATTATATATC	*apico*5715, *apico*6361, *apico*6832	5,415–7,054
6	R^*^: TATTATTTTACAATTAAAATAACCTAAAC		
	F^*^: CAACATATAAATTTAGGTACTATAGG	*apico*9003, *apico*9096	8,785–9,778
7	R: GAGGTTTATATCCAATATTAAAAGG		
8	F: CCAAAATTTATAATAAAGGTTCG R: ATCCAACTTCCTATAGGTTTA SF2: ACTAGAAGGGGTAAATGATA Sr2: TATCATTTACCCCTTCTAGT	*apico*11066, *apico*11619, *apico*11671	10,497–12,414
	F^*^: AATTCGATTGGCATTTCACC	*apico*26659	26,347–27,183
9	R: GGATTCATGCTCCGAAGGTA		

F, forward; R, reverse; S, sequencing. F and R pairs were used in PCR amplifications; SF, SR, and primers with

* *labels were used in the sequencing reactions*.

## Results

**Figure 3:**
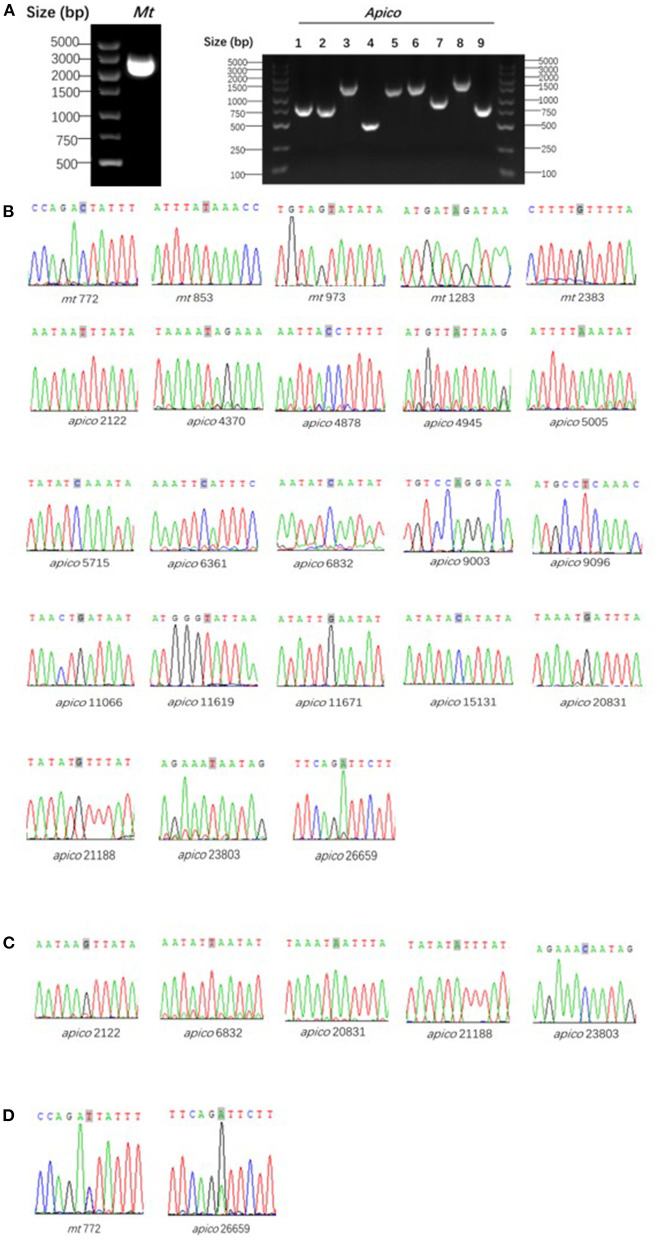
Agarose gel electrophoresis of polymerase chain reaction **(A)** and sequencing wave of the target loci with wild-type **(B)**, mutant type **(C)**, and mixed type **(D)** compared to the reference *Plasmodium falciparum* 3D7. Gray shading marked in the sequencing waves indicates the target loci.

**Figure 4:**
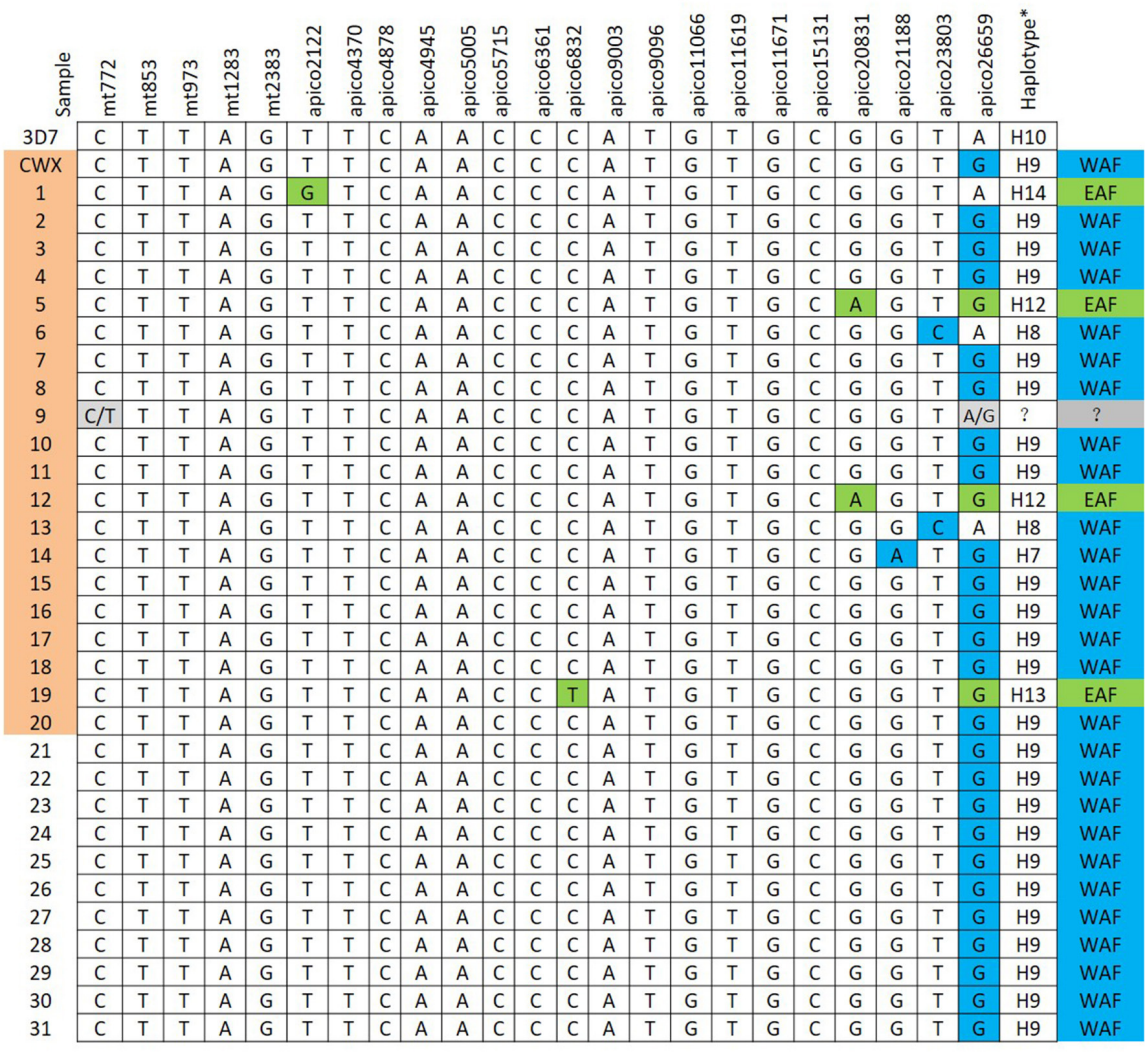
Geographically informative barcode of *Plasmodium falciparum* mitochondrion and apicoplast genome single-nucleotide polymorphism (SNPs). The sample sequence results from the 23-SNP loci were compared to the geographically informative haplotype barcodes previously defined (7). WAF, West Africa; EAF, East Africa. Sample information: 18: Guinea, 19: Republic of Congo, 20: Sierra Leone; others originated from Equatorial Guinea. CWX and 1–20 samples marked with color were isolates with *PfK13* mutation. *Haplotypes are based on the reference (7).

The reference *mt* and *apico* sequences were selected from the complex database and were shown to be well-correlated to the loci of the barcode designed (7). With the designed primers, the 32 isolates were amplified and sequenced successfully (Figure 3); however, the sequencing peak chromatograms showed one sample with a double peak at the two special loci indicated in mixed haplotype infections. For the five loci of *mt* genomes, including *mt*772, *mt*853, *mt*973, *mt*1283, and *mt*2383, the wild-type was shown except in one isolate with a double peak at *mt772* mentioned previously. A total of 18 loci of the *apico* genomes were selected for the barcode, with six loci including *apico*2122, *apico*6832, *apico*20831, *apico*21188, *apico*23803, and *apico*26659 with different alleles (Figures 2, 3). The haplotype analysis of *mt/apico* among all 32 samples in this study revealed seven distinct haplotypes. Figure 4 and Appendix 3 (Supplementary Material) show the number of haplotypes identified in samples from each country for the seven haplotypes identified therein. A single mutation at G_26659_ of *mt*/*apico* (haplotype 9) was the most prevalent haplotype (22/29) in Equatorial Guinea, which is unique to West Africa. In addition, haplotype 9 was also found in the isolates from Guinea and Sierra Leone. Among isolates from Equatorial Guinea, the C_23803_ single mutation (haplotype 8) was found in two isolates, and the A_21188_G_26659_ double mutations (haplotype 7) were observed in one isolate, corresponding to the barcodes published for West Africa. Meanwhile, the isolate CWX, with 23-SNP barcode identified in this study was consisted with the results analyzed with whole-genome sequencing (3).

The A_20831_G_26659_ double mutations (haplotype 12), the T_6832_G_26659_ double mutations (haplotype 13), and the C_2122_ single mutation (haplotype 14) identified in some of the isolates correspond to the barcodes published for East Africa. Haplotype 12 and haplotype 14 were observed in two isolates and one isolate from Equatorial Guinea, respectively. Haplotype 13 was found in only one isolate from the Republic of Congo. In addition, the mixed genotype of the wild-type and position *mt*772, *apico*26659 mutations, was found in one sample from Equatorial Guinea.

## Discussion

The Greater Mekong Subregion is the cradle of now widespread resistance to previous frontline antimalarial drugs (10). There is a significant risk that the artemisinin-resistant phenotypes within the Greater Mekong Subregion may similarly spread to other endemic regions. Therefore, the geographic origin of the parasites is important for monitoring the global emergence and spread of resistance and may prevent or delay the spread of artemisinin resistance from South East Asia to sub-Saharan Africa (11, 12).

With the establishment of sequence-based genome-wide polymorphisms in *P. falciparum* parasites, it is becoming feasible to design panels of SNP-based genotyping assays in tracing parasite geographic origin. Highly polymorphic microsatellites are useful to characterize community-, country-, or region-level genetic diversity over relatively short periods of time, as often required in outbreak investigations. However, it is difficult to standardize the interpretation of microsatellite assays across laboratories (13). The genomes of *mt* and *apico*, which have uniparental inheritance and do not recombine with each other, could provide more stable region-specific genotypes than nuclear genome SNPs. As a result, Preston et al. (7) developed a 23-SNP barcode using polymorphisms from *mt*/*apico* to identify the geographic origin of *P. falciparum* strains. In the study, 151 SNPs in *mt* and 488 in *apico* were identified from 711 *P. falciparum* samples in five geographic regions: West Africa, East Africa, Southeast Africa, Southeast Africa, Oceania, and South America, and a 23-SNP barcode was designed to trace the origin and dispersal of parasite strains across and between continents (7).

PCR-based approaches are inexpensive ways of extracting genomic data from samples containing very small quantities of parasite DNA, excluding the interference of host DNA. PCR-based approaches that genotype small collections of SNPs or a limited number of amplicons are of great value for timely genetic analysis of clinical samples collected directly from sporadic cases. In this study, we chose the method that used a 23-SNP barcode based on single-step PCR (ssPCR) and sequencing because it is a low-cost, rapid, and easy method to perform in the laboratory. This study presented the feasible application of PCR and sequencing in examining the 23-SNP barcode from *mt*/*apico*, effectively identifying the geographic origin of *P. falciparum* strains imported to China.

In this study, 10 fragments were amplified with 10 sets of primers by ssPCR, and the amplification efficiency of *mt* was much higher than *apico* (Figure 3A). Molecular targets are involved in malaria diagnosis assays; 18S rRNA genes are the most common; meanwhile, *mt* and *apico* are also an obvious target for malaria diagnosis (14, 15). Normally, the 18S rRNA gene of *P. falciparum* has 7-fold the amount of nuclear genomes, while *mt* and *apico* are ~20-fold and between 1- and 15-fold, respectively. All of them can be used to classify samples with mixed infections; however, ssPCR targeting 18S rRNA still showed similar sensitivity with targeting *mt* and even higher than the *apico* genome-based classification. Of the 23-SNP barcode, a total of 13 loci were polymorphic and composed of African haplotypes (7), and only seven loci were found to be polymorphic in this study. Then, it could also select specific loci for amplification and attempt to predict the geographic source. Our results confirm the relatively low efficiency of the amplification of *apico* targets. Although the limit of detection was not tested, we recommend using whole blood, high-efficiency enzymes, and DNA enrichment if necessary.

We not only supported a feasible method for the 23-SNP barcode assay, but we also listed the clear sequence references of the targets and genome of *apico* (Appendices 1, 2 in Supplementary Material). For the accurate demand of the assay, the reference sequences must contain the original design (7); even a single deletion/insertion will make the analysis change completely. The genome of *P. falciparum* 3D7 was the first reference genome published in *Plasmodium* research; however, the *apico* was not sequenced in the original genome project, and the *apico* from the *P. falciparum* isolate C10 was used (GenBank X95275.2, X95276.2) (9) until version 3.1 of the *P. falciparum* genome including a complete *apico* genome was uploaded (16, 17). We checked the database carefully and found X95276 (1–14,009 bp) combined with the reverse sequence of X95275 (14,010–29,430 bp) was well-confirmed with the loci selected in the design. Our sorted data supplied good reference for the later relative study.

In the present study, parasites were screened from origins that are not yet included in the network (WAF: Burkina Faso, Gambia, Ghana, and Mali; EAF: Kenya, Malawi, and Uganda) (7). Most of the malaria imported cases in this study were acquired in the central–western part of sub-Saharan Africa (Equatorial Guinea and Republic of Congo), and two isolates from West Africa were selected as control (Guinean and Sierra Leonean). Based on 23-SNP barcode analysis, West African isolates as well as the 25 isolates from Equatorial Guinea were confirmed to have the same haplotypes as those originating in West Africa. Surprisingly, the geographic origins of three isolates from Equatorial Guinea and one isolate from the Republic of Congo were East Africa. In addition, one isolate from Equatorial Guinea showed mixed haplotypes, indicating either heteroplasmy or multiclonal infections, in which it was difficult to infer the geographical origin for the parasites with multiple *mt* and *apico* genomes. The confused results consisted of the prediction that the 23-SNP barcode may lack the genetic resolution to distinguish between ongoing autochthonous transmission and malaria infections imported from one or more nearby locations (7). However, they are better suited to tracing the origin and dispersal of parasite strains across and between continents. With limited samples, this study still could provide the sequence data from Central Africa and fill data gaps in sample origins that are not yet included in the network.

Previous studies revealed the highly conserved structure of *mt* and *apico* in the genus *Plasmodium* (18, 19). The ratio of NS/S substitutions, which can be used to gauge the intensity and directionality of natural selection, are generally quite low for both organelle genomes, indicative of the strong purifying selection on NS sites, but see Preston et al. (7) and Wicke et al. (20) for exceptions to this trend (21). In this study, NS/S ratios were quite low for *mt*; this points to the highly conserved structure of *mt* in *P. falciparum*. In addition, *apico* genes had a higher NS/S ratio than *mt*, corresponding to the previously published study by Preston et al. (7). Pressure from antimalarial drugs may account for the high NS/S ratio of the *apico* SNPs. As translationally active organelles, *Plasmodium mt* and *apico* have been validated as important drug targets.

The *apico* genome encodes ~30 proteins, and most are ribosome subunits. Apicoplast has been identified in interactions with the delayed death phenotype caused by antibiotics, which may inhibit the *apico* housekeeping functions (22). Ribosomes of the *Plasmodium apico* and *mt* have been validated as targets for antibiotic action (23). It has also been reported that the artemisinin-exposed persistent forms restructured the mitochondrial–nuclear associations in *P. falciparum* (24). In contrast, rapid killing of malaria parasites by artemisinin is thought to result from depolarization of the mitochondrial membrane (25). The *Plasmodium mt* genome encodes only three protein-coding genes; however, each parasite has multiple genomes in *mt*, which may allow suboptimal mutant genes to preadapt to drug resistance even without strong drug-selective pressure. Studies into the mutant genes of *mt* and *apico* with drug pressure are rare; conclusive evidence is still lacking. In this study, the selected 32 isolates were from Chinese travelers who had returned from African countries. CWX has been reported as an artemisinin-resistant strain (3), and the 1–20 isolates have been confirmed to have the *Pfkelch13* mutant genotype. The other 11 isolates (21-31) showed *Pfkelch13* wild-type in another study. However, for the limited number of Kelch13 mutant samples, drug assays were not included and also out of the focus of the study. Multiple surveys of populations subjected to drug pressure are necessary to confirm the haplotypes and drug pressure.

In conclusion, this study provides a practical and highly valuable method to trace back to the geographic origins of *P. falciparum* malaria based on PCR and sequencing using the 23-SNP barcode from *mt*/*apico*. As population mobility has increased, the risk of reintroducing parasites to elimination areas and dispersing drug-resistant parasites to new regions has also increased, malaria control programs should be prepared to respond to this.

## Data Availability Statement

The raw data supporting the conclusions of this article will be made available by the authors, without undue reservation, to any qualified researcher.

## Ethics Statement

The studies involving human participants were reviewed and approved by The Institutional Review Board of Jiangsu Institute of Parasitic Diseases. The patients/participants provided their written informed consent to participate in this study. Written informed consent was obtained from the individual(s) for the publication of any potentially identifiable images or data included in this article.

## Author Contributions

FH and QZ performed the experiments and wrote the manuscript. GZ, HZ, and MZ helped with the sample collection and conformation. YLi and FT designed the experiments and analyzed the data. FL and YLiu conceived the experiments, provided advice on data interpretation, and edited the paper. All authors contributed to the article and approved the submitted version.

## Conflict of Interest

The authors declare that the research was conducted in the absence of any commercial or financial relationships that could be construed as a potential conflict of interest.
