# Supplementary material for: A PCR-Based Technique to Track the Geographic Origin of Plasmodium falciparum With 23-SNP Barcode Analysis
Source: Front Public Health. 2021 Apr 16;9:649170. doi: 10.3389/fpubh.2021.649170 (PMC8085391; doi:10.3389/fpubh.2021.649170)
Supplement: Supplementary file 1 [file Data_Sheet_1.docx]

**Appendix 1**

The reference sequences used in this study were from the database of Genbank (https://www.ncbi.nlm.nih.gov/nucleotide/), AY282930 (5949 bp) was selected as the reference sequence for *mt*, and X95276 (1-14009 bp) combined the reverse sequence of X95275 (14010-29430 bp) for *apico*.

>Pf_Api | Plasmodium falciparum 3D7 | X95276 (1-14009 bp) and the reverse sequence of X95275 (14010-29430 bp).

AATTATACCAATGATATATCAATTATAAAATTTTTTTGTAAAGTTGAGTAGTTAAGTATAAAATTTTAATTGTTGAAGTAATATAAATAAGAAAATTTAGTTAAAAATAATATTCTAATCATATAAATGAATAAAACAATAATTAGAAAAATTATTTTATAGGATTAGTATGTATTTATAGTAATATATTTAGTGTATAATTCCTAATAAGTTGAATATTTAATTATTTATTGTTATATTTGCTAAAGTAGCTTAATTGGTAAAGCAACTGATTTGTAATCAGTAGATTATGAGTTCAAATCTCACCATTAGCTTTTATTATTTTTATATATAATTATTATGATAAAATTTTTAAAACCTAAAATAAAAATATTAAAAAAATTAAATATACCTTTTTTATTATATTTATCTGGTAAATATAATTATAAATGTTTAAATTATAAAATTTCATATAAATCTTATTTTGATTTAAAATTAAAATTTATTAGATATATATGTTATAATTATTGTATAACATATAAAAAATATTTATATTATTTGAATAAAATAGATAATAAAAATATAAATATTTTATATTTTAAATTATTAAAAATATTAGAATTAAGATTGGATATATTTTTAGTTAATATAGGTTTTTTTAAAACTATATTGCAATCAAGGTATTATATTAAATATAAAAATATTTATATTAATAATATTATAAATAAATATTATAATATTAATTTAAAAAATAATGATATTTTATTTTTTAATAATAAAATAAAATATATAATATTAAAAAATTTAATTTATAAATATAATATTTATATTTACATATCTAATTTATATAAATATAATTTTATTAAAATATATAGTTATAATAAATATTTTATAATATGTATTTATAATTTTAAAATTAAAATATTAAATATAAATAATATATTAAATAATATATTATATATTTATAATGATATATATTATATATAATTAATAGTTTTTATGATAAATATAATCTAATGGTTAAGATGAAGAATTGTGGTTTCTTTTATATGAGTTCAAATCTCTTTATTTATCTGTTAAATATAAAAATTTAATGATATAACTTAATTGATAAAGTAAATAATTGCAAATTATTATATTTCAGTTTGAATCTGAATATCATTTAAAGAGAGATATGGTGAAATTTGGTATACACAATGGACTTAAAATAATTTGAGTTAATTATTATTAATATTAAATTTTTAAGAAAATATATAATATATTTTTTTAAATTCTGTAATATATTTTAAAATTTATATATTCAAAAGACTTTATTTATAAAAAGCTAAATTTATTAAGAAAATCCATTAACATTATTGTTGTAAGGGTTCAAATCCCTTTATCTCTAACTATAACATTTATAGCTAAGTGGTCGAAAGCAATGGACTCATAATTCATTTTCAATATATTGATCATCAGTAGTTCGAATCTACTTAAATGTAATTTAAAGTTAATGCCTGAGTGGTTAAAAGGAATGGACTGTAAATCCATTGATAATATATCTACATCAGTTCAAATCTGATTTAACTTATTTTTATATATAAAGAGAAATGACTGAGAGGTTTATAGTTATAAATTGCTAATTTATTGTATATATAATAATATTATACCAAGGGTTCGAATCCCTTTTTCTCTATTATTAGAATTTGTTAGTTTAATTAGGTAAAAATATTATTTTGTCATAATAAAGAATACGAGTTCAATTCTCGTCAAATTCGTTTTATTTAAGAATTACTAGCTTAATTGGTAGAGTACTCGACTTTTAATCGAATGGTTCTGAGTTCAAATCTCAGGTAGTTCATTTAATAACTTTTATCGTTTAAAGGTAAGACATCTTTTTTTCAAGAAGAAAATAGGAATTCGATTTTCCTTAAAAGTATTTATTTAATTCAGAATATAGTGTAATGGTAACATATCTATTTTGGGAATAGAAGAATATAGGTTCAAATCCTATTTTTCTGAAAATAATATTATATGTATATTTTAGAATGAATATTATTATTTTAAATAATAATACATTAAATAATATAATATTTAAATATAAATATAATTTTTTTATTAAATTATATTTTAATAATTATATTAAAATATGTAAATTAATAATTTATATTATAAAATATTTATATATATATAATATTTATATGTATAAACATACTAAGAATAAAAGTAAAGTATATTTTAGTAATAAAAAAATAAGAGTACAAAAAGGGCTAGGAAAAGCTAGATTAAAAAATTTTAAATCACCTGTATGTAAACAGGGTGCTTGTAATTTTGGACCTTTTTATAAAGAAAATAAAATAATAAGTAAAATAAATTATAGATTAATTTTTGTTTATTTATTAATAAATAAACGTAGTAATATTATAATAATTAAATTAGAAAATATTATAAATTTATTAAATATATTTTATAAAAATAAAAATTATTGTATATTTAAATTATTATATTTAAAAGGTATAATTAATAATAAATATATTTTAATTAATTTAAATAATAAATTATTTAATAAAAATATATTTATAAATATTATTATGTATAATTATTTAATATTTTTAATTTAAATTATGAAAGAAGTTATTTTAAATTTTTATTTATATAATATTTTATTTTATAAAATTAATGTTTTAAATAAATTTTGTATTATTTATTCTATAAAATATTTTACAAAATTAGATATAAAATATATAATTAAAAATATATTTAAAATAAAATTAATTAATTATAATAATATAAAAATTAATAATATAAATAAAAAAAATTGTTTAAAAAAATATTATATTACATTTAAATGATATTAAAATTAAAAAAATATAATACTTATAAATATTTTAAAAGTTTTGGTAAAAATAATAAAGGATATATTACTATTTATAATAAAGGAGGTGGAAATTTAAAATATAATTATAAATTAATAGATAGTTGGTATGATAATTATAATATTACTATAAATTATAAAGTTTTTTTATTTAAAAAAATAAAAAATTATTTTAGAAATACATATATAGGATGTATTTTGTATTTATCTAAATTAAATAATTTACAAAAATTTATTATTTTACAACATAATTATATGATAAATTCTATATATTATTTAACAAATATTAATAATATTAAAACTGGAAGTTATATACAATTAAAATATTGTAAATTAGGTACATATATCTATAATATATCTAAAGATTATAAAAAAGGTAGTATTTTTGCTAGATCAGCAGGTACTTTTGCTCAAATATTATCTTTTTATAAAAATTTAGTTTATATAAAATTACCTTCTAAACAATGTAAATATATAAATATAAATAATTTTTGTTATATTGGTGTAAATAGTAATATTTTTCATAATAAATTTAAAATAAAAAATGCTGGTTATAATATTTATTATAATATTAAACCTAAAGTAAGAGGTAAAGCTAAAAATGTATGTGACCATCCTCATGGGGGAGGTAAAGGAAAGACTGGTATTGGTCGTAAATATCCTTGTTCTAAAAAAGGATTACATTCAAAAGGATATAAAACAATAAAATAAATATAAAAAAAAAAAAATTAATGATAAAATTATATTGGTTAAAAATTACTATAAATAATAAATATATATTTATAAATATAAATAAATATAAATATAATAAAAATTTAATTTTAAATATATATAATAAAAATTTATATATTTATAAAAAATTATTAAATTTATATATAAAAGTATATAATGGATATAAATTTATACCTATTTATATAAATAAAACTAAGTTATTTAATAAATTAGGAAATTTTATATATACTAAATTTGTAAAAAATAATATTAAAGAATTATTAATAAATTAAAAAAAAGATATGGGACAAAAAGTACATCCTTTAATATTTAGAGGATTAATATATAAAAACTATTTAAATAATTTTTATATAAATATTAGTAAAAATAAATATTATTTAATTAATATATTATTAACATATTTTATTTATTATAATATGTATAGAATATGTAATTATAAAGAAGATAATTATATTAATATAAATATAAATTTTAGTATAAATAAATTTGTAATTACATTTTTTTTATATAATAAATATTATAATATATATAATTTAAAATATATATTTATTTTATTAAATTATTTTAATTATTATTATTATAATTATTATAATTATATGTGTTTTTTAAAAATAAAATATGTAAATAATATAAATAATATTAATGTTATAATGTATTATATAAAAAAATATTATATAAAATATAAATCTTTAAAATTAATATTTGATTATTTATATAATAATATTTTAAAAAAAAATAATTTTAATATAAAAGGATTAAAAATTAAATTTTCAGGTCGTTTTAAAAATAGTTTAAAGACTAAAATAGAAATATATATTTATGGTATTATATCTTTAAGTACATTAAATAATAATATTAAATATATAAATGATATTATAAATACTAAATATGGTATTTTAAGTATAAAAATTTGGTTAAATATTTAAAAATAAATTAAAAAAATTTATAATATATGACTAATATTATAATAAAAAAAAATCAAAAAGGTAAAATAAAAGGTAAATTTAATTTAAAATTTTTAAGTTTATATTGGGGTATTATATCTTTAGATTCTGGATTTTTAACAAAAAATCAATTAGAGACTTCAAAATTTATAATAAATAAATATTTAAAAAAAATAGGAGTATATAAAATTTGTATAAGATGTATAAAATCATTAACTAAAAAATCTTTAAAAACTAGAATGGGATCAGGAAAAGGATCTATAGAATTATATGTAAGTCCTATAAAAAAAAATAAGTTATTATTTGAAATAAGTAAAATTTCAAATAATATTATTTATACTATAACAAAAGTTTTATCTTATAAATTACCTTTTAAATTACAATATATTAGAAAATAATTAAATAAAGAATATGAATATAAAAATAGGATATGTTATTAAGAATTTAAATATAAATATTAAGATAGTTTGTATATCTTTTTATAAGTATAATTTTAAATATAAAAAATTATTATTATGTAATCTATATATAAAAATATATGATAATAGAAATGAAATTATTATAAATGATTATATATTATTTAAATATTATAAAAAAAGTAAATATTGTAATAATAAAGTAATAAAAATTTTATGATATATATAAATAGTATATTAGATGTAATAGATAATAGTGGTATATTTAAATTTAAATATATTTGTACTTTAAATAAATATAAAAATCCTAAATATGGTGATATAGTTATAGGAGTAGTTTATAGTTTATATAATAATAATTTATATAAAAAATCAGATAAATGTAAAGGTATTTTAGTACAACAAAAAAAATTTTTAAATTTTAAAAAATATTATTCTATAAAATTTAATAAAAATGCTGTAATAATTATAAATAATAAGTTAAATTTTGTAGGTACTAAAAGTAATAATTATATATCTAAATATATAAAATATAAATTAAATATGAATAAGTTTAAATAAATTTATTTGAATTATGATTATTAAATTTTTAAATAATGTTAAATATAATTTTAAATTAAAAAAAAATTTTATATTATATAAATATAATAAAGTAATATATTATTTAAGTATATTATTATATAATTATAAATATATATTTAAATTATATATTTTAAATATATATAATAAATATTTTATTTTTATAATTTTAAATGAATATAAAAATATTAATTATTTTAAAGTATATATCAAATATAATCAATTATTTTATATGAATTTTAATAAATTATTATGTTTTATAAAATTTAAAAAATATTTTAAAGGTTTATTAATTTTATATTCTTCAAAATATAAATTTATTACACATATATTAGCTTTAAAATATAAAATTGGTGGTATTTTATTATTTTATATTTTGTAAATATTATAGTTATAATTGTTATGATAAATAATAGAAAGTACATATTTTTAAATAATAATATAAAAGAAGGTAATATATATTTAATTTTAGATATAAAATATTTGAATTATATATATATATTTAAAAATAATAATAAGATATATAATATTAATATATTAAATAATATATATATATATTTTATAAAAAATAATATTTATTATTTTATATTTAAAATGTATAAGTATATATTTAGTAATATATTTAATAAAATACAATATAAAAAGTATCAGTTAATTTTAAATATAATTGGTATAAATTATAAATTTTATTATATAAAAAAAGGTAATTTTTTAATATTTCAATTAAAATATAGTCATAAAATAATTATTAAGTTACCTAATATAATATTTTGTGAGTTAGATATAAATAAAAATTTTATATATTTATATAGTATGGATATATTTATATTAAATTCTATTGGGAATTTAATAAATTCATTTCAATATATAAATAAATATAAAGAATTAGGTATAAAAAAATTAATATGATAATTAATATTATTAATAAAAAATTAAATAATTTTTATATATTATATTATGTTATAGTATTATTTAAAAGTATTTTTATATTAATTTTAAAAAGAAAGTTTAATAATTATTTGTATAAAATTTATAATATTTTAATTATATATTTAAAATTATATTATATTATAAATAATAAGAGAGATAAAAAATATATTTTTAATAATAAAAATGTAAATTTTATTTATTTTTATATTTATAAATATAATAATAATATTAAGTTAAATAATAGTATTATAAAGTTAAATAATAATATTATTGAAAAAATTATTGAAATAAAAAAAATATCTTATACAATAAAAAAAGGAAGAATTAGAAGATATAAAATAGTATTAGTATTAGGTAATAAACAAGGTTGGATAGGATTAGGAGTTAGTAAGAATATCAATATTAATAAAGCTATTATATCTGCTAAAATAAAAGCTTTAAATAATATTTATTATTTTAAATATTCTATATTAAATATATATAAATTAAGATATATATATATTAATTATAGTAAATTTTTTATTAAATTACAATTTAAAATTTATAATTATTTAAATATAAGATTTTTATTATTAAAATATTTATTTGAATGTTTAGGTTATTTTAATTGTAAAATAATAATTTATTATAATATAATACATAATAGATATAATTTATTAAATAAATTATTATTAATATTATTTAATATATTTTAAAATTAAAATGACTTTATATTTAAATAAAAATTTATTTATATATATATTAAAATATATATATGGGTTAAATTTATATAATATTAATATATTATTAATGATAAATAATATAAGTATATTTAATATTAAAGAAAATAATATCAGTAATATTAAAATTAGTTTATATAGATTAAATATTTATTTACATAAAATTTTAAAAATAAAAAAAAATAATATAATTATAAATAAAATAAAAAAATTTAATATAAAAAATGAAAAAACGTTCTTCAATAAAAAAAATATGTAATAAATGTAAATTAATAAAACGTTTTAAAAAATTACATATAATTTGTATAAATAAAAAACATAAGCAAACACAATGATTAAATATAATAATTATATAATTTTTTTATATTTTAAATTAACTTTAAAAAATACTTTAATAACAATATCTAAATATAAATATAATAATAATAAATATATTTATATAAAAAATATTAAGAATATATCATGTGGTTGTTTTAAATATTTTAAAAATAGATTAAAAAATACTATATTAGCAAATAATATTTTAACTATAAATATTATAAAATATTTAATTAATAAAAATTATTTAAATATAAATATAATATTTAATGGTATAAATTATTATAGAATACATATATTAAAATTATTATTAAATGTAAAATATAAAAATAAAAAATTAAATATAAATAAATTATTTGATATAACTTCAATACCTTATAATGGATGTAAATATTCAAAAAGAAAATATTAATAAGATATAAAAAATGTTAACAATAAATAAATTATTATATAAAAAAAAATATAAAAAAACTAAAAAAATAACAAATTATTTATTAAATAAATGTCCTCAAAAAAAAGGTATAGTTTTAAAAATATTAATAAAGACTCCAAAAAAACCAAATTCAGCTTTAAGAAAAGTTGCTAAGATAAGATTATCAAATAATAAGGAATTATTAGCATATATACCAGGTGAAGGTAAATCTATTCAAGAACATAATTTTGTATTAATTAAAGGAGGTAGAGTAAAAGATTTACCAGGTATAAAATATAAAATAATAAGAGGTTCTTTAGATTCAACTGGGGTTTTAAATAGAAAAACTTCTAGATCTAAATATGGAACTAAAAAATATTAAAATATATAAATAATAATGATAATATTTAAGTATTTTATAAAAATATTTTTGAAAAAAGGTAAGTTAAATAAAAGTATAAAATTATTAATATATATATTATATTTATTAAAAAAAATAACTAATAAATCTAGTATATTTATTTTTAATAAAGCTATAAAAAATTTATTATTACCTTTTTCTTTTTTAAAAATAAAAATAAATCATATTAAATATAATATACCTATTATAATATCGTATGAACAATCTATATTTAATATATATAAATTATTAAATAATATTATAAAAAATAAAAATATTTTACTATATAAAATTATTTGTAAATATATAATTTTTAGTTATAATAAAGAGGGTGAATTATATAAGATAAAATTAAATTTAATTAAACAATTTATATCAAATAGAGTATATATATATTTATTAAAGAAAAATAAAATTAAAAAGTGATTATATTATTATTTATTATTAATGAAATAATATTTTAAATAAAATATGAATAATAAATTATTTTTAAGAAATAAACAACATATAAATTTAGGTACTATAGGGCATGTAGATCATGGAAAAACTACATTAACAACAGCTATATCTTATTTATTAAATTTACAAGGATTATCAAAAAAATATAATTATTCAGATATTGATTCAGCTCCAGAAGAAAAAATAAGAGGTATTACAATAAATACAACACATATTGAATATGAAACTTTAACAAAACATTGTGCTCATATAGATTGTCCAGGACATTCCGATTATATTAAAAATATGATTATAGGAGCCACACAAATGGATATAGCAATTTTAGTAATATCTATAATAGATGGTATAATGCCTCAAACTTATGAACATTTATTATTAATAAAACAAATAGGTATAAAAAATATAATTATTTTTTTAAATAAAGAAGATTTATGTGATGATGTTGAATTAATAGATTTTATAAAATTAGAAGTAAATGAATTATTAATTAAATATAATTTTGATTTAAATTATATACATATATTAACTGGTTCAGCATTAAATGTAATAAATATAATTCAAAAAAATAAGGATTATGAATTAATAAAATCTAATATTTGGATACAAAAATTAAATAATTTAATTCAAATAATTGATAATATTATAATACCTACTAGAAAAATTAATGATTACTTTTTAATGTCAATAGAAGATGTATTTTCTATAACAGGTAGAGGTACAGTAGTAACAGGTAAGATTGAACAAGGATGTATAAATTTAAATGATGAAATTGAAATTTTAAAATTTGAAAAATCATCTCCTAATTTAACAACAGTTATAGGATTAGAAATGTTTAAAAAACAATTAACACAAGCACAATCCGGAGATAATGTAGGTATTTTATTAAGAAATATTCAAAAAAAAGATATAAAAAGAGGTATGATTTTAGCAACACCTAATAAATTAAAAGTATATAAGTCTTTTATAGCTGAAACATATATTTTAACTAAAGAAGAAGGTGGTCGTCATAAACCTTTTAATATTGGATATAAACCTCAATTTTTTATTCGTACAGTAGATGTTACTGGAGAAATTAAAAATATATATTTAAATGAAAATGTACAAAAAGTAGCTATACCTGGAGATAAAATAACATTACATATTGAATTAAAACATTATATAGTGTTGACATTAAATATGAAATTTTCTATTAGAGAAGGAGGAAAAACAATAGGAGCAGGTATTATAACAGAAATAAAAAATTAAATAAAATAAGATGAAAAATATAACTATTAATAAATTTTTTATAAAAAAAATATTTAAAATTATTAAATATTTTAATATAATTTATTATCGTTTATTTATTTGGATATATATTATTATTTTATTATTTATTTTAGTTAATAAAAAAAAATATTATAATACTATAATATATAACAAATATAAATATTTATTAAATTTTTTATTTATAATTTTATTATTAAATAAATGTCAAAAATCAGATTTGAACTGATAACACATGGATCTTCAATCCATTGCTCTACCATTGAGCTATTATGACTTATTATATATAATAATATATATAGAATATAACCAAAAGGTTAAGGTAATGAATTTTGATTTCATTAATATAGGTTCGAATCCTATTATTCTAATAATAATGAATATAATTTAATGATAAAATACAATTTTACCATAATTGTTATAAGAGTTTGAATCTCTTTATTCATATATATAAAAATTATGTCTTTAATTTAAAGTAAAAATATAAATTTCCAAAATTTATAATAAAGGTTCGAATCCTTTAGGACATGTATATAATAATATATAAATAAAAAAATGATTATTTCATGTAAATATAAATTTTTAATAAAAAATAATCAAATAAATTTAAATTCAATTTTAAATTTTAAATTTAAAATATATAACATTAATATTAATTTATTAAATAAAAAAATAACAGAATATATAAATAAATATAAATTGAATTTATTTATAATTTATATTTATTCAGACAAAACATTTAAAATTATATATAATTATACAATATATAATTTATATAATAAATATAATAGTAAAGTAAATAAAATATTACTAATATATAAGATATTATTATATAAAAAATTTCAATTATTATTTTATAATATTAATCAATTATTATATATTATAAAAAATAATTTTAAACAAATAAACATAAAAAATAATAAAAATCATGATAATTTTAAATAATCTTTATTGTACAAAAGAATTAATAATAATATTTATTAAATCTGAATATTTAGCATTAAAATATAATAATAATTTTATAATGCCTATTCATTTATTATTAGGATTATTATTAACTGATAATTTATGTACAAAATTTTTAAAAATAAATAAAAAAATAATAAATAATAAAATAATTTTATCTTTATTAAATAAATATAAATATAATAATAAAAATATTATTAATATAAATTTTTCTAATAAAGTTATTAATATATTAATTAAATTAAATAATTTTAATTTTAAAATTAATTCATTTAATTTATTATTATTATTATTAGAAGAAAAAAATAATAATAAAGATATTAATTATTTATTTAAATATTTAAATTTAAATTTTTCTAATTTAAATTTAAATAATTATATAAAAACTAATATTTTTTCAAATAATATTAGAATAAAATTAAAAGAAATATCAGTAAATCTTTTAAATTTAAATTATATTTATAATAATAATTTAAATTTTTATAAACAACAATATATACAGTTATTACAAATTTTAAATTTAAAAATAAAAAAACATATAATACTAGAAGGGGTAAATGATAATATTTTTATATTTTTACAATTATTAATTAATAATATAAAAAATAAAATTATACCAATATATTTAAAATATACAGAAATATGGGTATTAAATGATTTATTAACTTATGATATACAAACTTTAATATATAAAATATTGAATATATCTAAATATTTTACAAATAAATATAAATTAATCTTAATTATAAAAAATATAGAAATATTTAATCTATCAGATAATATTAATAATGATAATAATAAATTATATTATTTATTTTTATTATTAAATAAATTATATGGATATAATATACATATAATAATAGTAACTAATAAAAAAGAATATAATACATATTTTAAATATAATATAATAAAAGATTCTTATTTTTATAAAATAAGAATAAAAGATTTATCAATATTACAAACATTCTTAATAATAAAAAATAATATATATAAATATATTAATTATTATAAAATTAATATTAATAATTATATTATATATGAATTAATAAATTTAAGTAAAAAGTATATAAAACCTTTAATATTACCTACAACTCCATTAATTTTATTAGAAAATTCATGTTCTAATAAATATTTATTAAATAATAAAATATCTTATTCAAATTTTAATTATTTATTTACATATAATAATAATATTATATATAATAATAAAAATAATAATTTAACTATAGAAGATATTAAAAATTCAATATCTAATTACTTAAATATATCTAAAACCATATTATTTAAAGACAATAAATTAACTAAATTAAATTTAACTAAATTAGAAAATTATTTATATAATCATATATATGGTCAAAATCATATTTTTAATAAAATAATACCTTTTATTAAACAAAATTTTATAGGATTAAAAAATAAAAATAAACCTATAGGAAGTTGGATTTTATGTGGGCCTAGTGGTACTGGTAAAACTGAATTAGCAAAAATATTATCAAAACAATTATTTGGTTCTGAAAAAGAACTAATTAGATTTGATATGAGTGAATATATGGAAAAACATTCTATTTCTAGATTAATAGGTTCACCTCCTGGTTATGTTGGTTATTCAGAAGGAGGTCAATTAACAGAACAAGTTTATAAAAAACCTAATTCAGTAATATTATTTGATGAAATAGAAAAAGCACATCCTGATATATATAATATAATGTTACAAATATTAGATGAAGGTAGATTAACAGATTCTACAGGTAAATTAATAGATTTTACACATACAATAATTTTATTAACAAGTAATTTAGGTTGTCCAAAAAATTATGATTTATATCTAAAAAATAAAAATTTTTTATCAAAATCGGATTTAAAAGAAATAGAAAAAAATATAAAAATAAATATTAATAATTATTTTAAACCTGAATTATTAAATAGATTAACTAATATATTAATATTTAATCCTTTAAATATTAATAATTTATTATTTATATTTAATAAATTTATAAATGAATTGAAAATAAAATTATATTTAAATAAATTAAATATTATTATACATATTAATAAAGAATTAAAATATTTTTTAGTTAAATTAATGTATAATCCTTTATATGGAGCTCGTCCTTTAAAAAGAATATTAGAATTAATTTTTGATAAATCTATAAGTGATTTATTATTAACTTATAATAAACATTATTTTATAAAAAATAAATATATTTTATATTATTATTTAAATAAATATTATAAATTAAATTTTAATATATATTTATTATAAATTTTTAACAAATATAGTTTAATCGGTAAAATATTAATTTTCCAAATTAATGATATGGATTCAATTTCCATTATTTGTATATATTTTAAATTATAAATAAATAATATGAAAAATATTCATTTATATTCAAAATCAAATAAAACGAAATATAAAACATATAAAATAAATTTAAATATAAAAAAAATTAAAAAATATAAAAATATAAAATTAGGTATTTATAATCCAAAATTAAATATAAATTCTTGCTTATATTATTTATTATTAAAATATTTAAAATATAATTTTAAATTAAGTAAAAATTTATTAAAACTTTTATTATATAAAATAAAATTATTATATAAATAATAAGAGAAATGACAGAGTGGTTTATTGTGTTTGATTTGAGATCAAAAAAATATAAATATATTTCATGGGTTCAAATCCCATTTTCTCTTTTATATTTTTATGTATTATTATAATGATATTTAATTTATATTATAAATTGAAAAAAAATTTATTATTAAAAAAATTTAAAAATATACAAATAAATAATAATATTAAAAAAATTGTATATATTAAATTATTTAATATATTATTAAAAAGTAAAAATTCAAATAATTATATATATAATATTATAAATAATAAATATAAAAATATAAAATTATTATATATTCTATCAAATAATAAATATAATTTATTATTATTTAAAAATATTAATTTATGGAATGTATTATTAAATTATAATATAATATTTAATAATATATATATAATTAAAAATATATTTACATATTTTTAATTACATATTTTTAATTTTACTATGAATTAATGAAGTATAAATTATTTTATATATTAATTTTAAAGATTTATAATTATTATAATTTCCTAAAATTTTTATATTTATATTACTTGAATCTAAACTTAAATTTATAAAACTTATTAAAATTAATTTTAATTTTAAAATTTCTTTTAAAATTAAATTTTTATTAAAATTTGTTAAAAATATGTATTTAGGTAGATTTATCATATTTTTTATACCATTAAATTTATTATATAATTTAATATAAATTATATTTAAATTATATATACATTTTTTAGATAATATATTATTAAAATATTTATTTTTAATTATTTTACTTATCCAAATATATATTATAATTTTTTTTTTTAATACAAACCAATTAGTTAATAATCCAGAAACCCATTTATTTATATATAAATTATTTGTTAAATTACATATTTTAATTGTAAAATTTTTTATTAAATTATTATTATTAATAAATAAAATTTTATTATTCATTAATGATATATTATAAATATATAAATATAACTTATATAAATATAAAGCTATAAATGTAAAATTTAAAATACAATAATTAAATTTTATTTTATATATATATTTATAATTATCAAAATAAATATTCTTATAAATATTTCCTATATAAATTTTTGATTTTAATAAATTATCAAAAGTAATAAACATATTTTAAAAAAATTAATTTACTAAATATCTATACCATCCATTACCAACAGGTAATAAATCAGTCAATATTATTTTAGATTTTATATCTATTAACCAATCAATTTTATTATTTAAAATATTTAAACTTATTATTTTAAATGTATTCTGAAAACTTATATTTGTTAAAAATCCAGAATTAGCTAAAATTGATTTTGTAATACCTAAAATAATTGGTTCATATTTATAAATATAATGTTTATTTAAATTTAAAGAATAATTAATTATATTTATTAATTGTAAAGATATTATATCATTATATTTAAATATTTTAAAATTATTAGATATTATCTTTATACATGATAACATTTTTTTTATAATAAGTTCAAAATAAATAGAAGGTAAATAAATATTTTGATAACTATATTGTTTCAAAATAGATTCAATTAAAATATTATATACATATATATATGAACTTTTAGTTGCTTGATAAATATTTATAGATTTTAATAAAAATCTAAAATAATATTTTAAATTTTTATTAATTGAATAAAATTCTGTATGTAAAGAATATCCACTATATAAAATAGAACTAATATCTTCAAAAATATAAGAATAAAAATTTAATTTATATTTATAATGATTAATATTATATTTATTACATATATTATATATATATATAATATTATTTAAATAATTATAATATTTTATATAAAATATTACATAAATATTATTTGAAATAAAAAAAATATTATCTTTTATATTTTTATTTTCAAATATTATATTTATAGATTGTAAACCTATAGTAATATCATTTAAATATAAATTATAAATATTATTATTAATATAATTATAAAATAATTTATTATATAATAAATTATTATTTATTAAAATATTTTTATTATATTGATATAAATTCATATTATTATTATAAATATAATAATTATTTTTATATATAATATGATTATGTATAAAATTATTATTATTATAAAATAAATTTTTTATAAAATATAAATTAATATTTATATTATATTTATATAAATATTTAATATAATTATTATATATAATATATAAATTATATTTATTATTTAACAATAAATATTTATACCAATTATATTTAAATATTTCATATAAATATAAAAAATTATTATATTTTTTTATAAAAAATAATTTATTATAAGTATACTTATAAAAATTTAAATATTTTTTAATTATAATATTATTTATAATATTTAAATATATAAATAATTTTTTTTCATATTTATAATATATAAATATATTATTTACATAAAATAATTTATTTAAAATTTGTATATTATTAAAATTTTTTAATATAAAATTCATTTTTTAAAATAATAATTTGAATTAATAAAATTATAATTATTATTATAAATTTTATAATAATAATTTGAAAATAAATTAAAATAATTAATTAAAAAATATATAACATTATACTTATTATTATTATTATTTAAAATAATACCTTTATTATATAAATTATAAAACTTTATATGATAATAATATAAATAATATGTATAAATATTATATAGAATCCATTTATTATGGATATTTTTTATTAATAACTGTATATTACTATAATTATAATATTTAATTACATTATTTAAATATTTAATAATAATATTATTTAAATTATATTTAAAATTCTTAGAAATAGAATTATAAATAAAATTACATTTTATATATTGATTTTGTAAAATATATTTATATTCAAATAATATATTATTATAATTATATAATATTTTATTCATTAAAAATATTAAATTAAATTTAATATTAATATATTTTTTAAAATTTATAATTAATTTAAATATTTTATTTATATTTAATTTATATAAATATATTTTATAAATTAAATATTTATTAAAATTAAATTTATCTTTTAAAATTGAACTAGCATGAAAAGTCCTTAATACCATTTGAGTACTAGGTTCACTTATAGCTTCACTAGATATAACTCCTATATGTTGTCCTAAATTATATTTATATAATTGTTTATAATTTAAACATGTATTACATATATTATTATATATATTACATAAATATACAGATTTAATATTTAAATATATATTTCTTCTATTATATAAATTTAATAATTTATTTAATATATATTTAGTTATATAAGTATTTTTTGTATAAATAAAAGTACCATTATTTAAATTTAAAATATTATTTTGTAAAATTTTAAATCTTAATATATTTAAAGGTAATATAATATTACCATATATATCCATATTTAATATATATTTTAATATAAAAGGTGATTTACAATTTAATTCTTTTATTATAAAATTACTTGTAATATTTATTAAACGTTTTGTTAAATATCCTGAATCTGCTGTTTTTAAAGCTGTATCAATTATACCTTTTTTAGATCCATAACAAGATAAAATATATTCATATATATTTAATTCATTTATATAATTATTTATAACAGGTTTTTCATAAATCATTCCTTTTATATTAGAAATATAACCCTTATAACCTATTAATTGTTGTAATTGAGAATATTTTATTTTTATTTTATTATTAAAAAATAAAAATAAATTTGAATAAATAGGATTAATTTTATTATATAAATTATTATTTAAAATACCTTGAATTTTATTTATAACTTTTAAATAATAATAATTATTTAAAAATACATTTATATAATTATTTTTAATTTCGTAATATTTATTATTATAAATATTATTAATTTTATTTTTATATAAAATTAATAAATATATAAAATTTGAAAAATCTTTTATATTTAAGGAATAATTATATAAAAAACTATATTCATATCCTAAATATAATAATTCATGTAATATTTTAAAACTTATATTATATTTAAATATAATTAATAATTTTTTTTCTAAAATTTTTAAATTATATTTATTAAAAAAATAAAAATACATATTTATTATTAATATATATACATATATAAATTTATAATTATTCTATTAATAGAAGTTAATATATAAAATATATTATTATTATTTATATATTTTATCCAAACTAAATTAAATATAAATAAAATATTATTATTATAATATTCATATATTTTTTCAATTGAATTAAAATAAAATATATTTAACTCATTATTATAATTTAATATTAATAAAGTATTTATTCCTAATTTATAATATTGTAAATTACTAAATAAATTTTTATTATTTGAAGGTGATATTATATTTTTATCAAAATTTAAATTAATATTAGATTCAAACTTTGAAGTTTTAATTAATGGTAAAAAAATAGACATTTGATCCCCATCAAAATCTGCATTAAAACTTGTACATCCTAATGGATAAAATTTTAATGAATAACCTTCAGTTAATAAAGGTTTAAAAGATTGTAAATTCATTCTATGTAATGTAGGAGCTCTATTTATAATAATAAATTGATTTTGTAATAATCTATTTAAAAATTTTTGTATAATAAATAAATTTTTATTAATTAATAAACTTTTAAATATTATATTTAATTTACTATTATATTTTAATATATTTATTAAAAAAGGCTTAAATAAATTTATACTAATATAATAAGGTAATCCAATATTATTATATATAATACTTGGATTTACAGTTATAACAGATCTACCGGAAAAATCAACTCTTTTACCTAATAATTTATATTTAATTGTACTATATTTACCTTGAAAAGTTTTACTAAAATTAAAAAAAGTATTATTATTTTTTAAAATTAATTTATTTATCAATAAATAATCAATTAATTGTTGTAATAATCTTTTTTCTATTATTTCAAATATAAAAAAAATATTATTACGTAAATATAACCAATATTTTAATTTATTATTTTTTAAAATTATTAATCTATAATTTTCATTTATAGTAGATATAATATATGTACTATTATTTATATAAAAATAAGGTCTTAATCCTGCAGGTAATATTGGTAATAAATCTAAAAATATCCAATTTGGTTTTATATTATTTAATATAAATAAATTAAATAAATTAATTTTTTTATATAAATATTTTTTATTATAATATTTATTATTAATTAATAATAATTCTTTATTATTTAATAATTCAGTTAATAAATTTATATTTTGTAACTTTTTATATAAAATATTATGAGAAAATAAATATTGAATAATATTTTTATATTTTTTTTTATATAAATTTATTTTTGAAAATAATTTATTATAATAAAAATACTGCTTATATTTAATATTACTAAAAAAATATTTATAATAAATTAAAAATTTTAAATAAAAAACATTTTTATTTAATAATAATGAAGCTACTTTTAAAGGACCAGTTAAATACCATAAATGTAAAATTGGAATATTTAAAAATATAAAACCTAATTTATATTTTCTATTTATATTTATTATTAATTTATTTTTACAAAATTTACAATATAAAAAAAATGAAAAATTATTTATATTATACATTTTTTTATTACAATTACAATTCCATTTATACATATAATCAAATATTTTTTCACAAAATAAACCATTTAAAATTGGTAATCCTGTATTAAAATTTATTGTATTAGGTATTAATACCTCTCCGATTATAATTTTATTTTTATAAAATATTGAAGACCATTTTATTATTTGTTTAGGATTTAATATATTTAATTTTAATCCTATAAAATTTATATTATTATGTATTATCATTATTAATAAATTATATTAATAGGTAAATTTTCTAATAAATTATTTGTATCATTAAATATACAAAAAGCTTCTATATTAATAGCTAAACTTTGTAACTCTTTTAAAATTAATTTAAAAGTTTCTGATATAAAAGTATTTTTTATTTTATAATTATTAAATAAATAATTTTTTAATATTTTTCTACTTTTAATATCATCAGATTTATAAGTAAAAAATTCTTTAAATAAATAAGAAGCTCCAAAAGCTTCTAAAGCCCATACTTCCATTTCTCCAAATCTTTGACCCCCTTGTTTTGTATTACCTTTTATTGGTTGTTGAGTTAATTCAGAATATAAACCTATAAATCTATATCTAAATTTATCTTTTATCATATGAATTAATTTATAATAATAAATATTATTTAAACAAATACTATTATTAATCATATTACCAGTAAATGGATTTTTTAAATAATATTTATTATAATTATAAGACATTTTACTATTTATATTAAAATTATTATTATAATTATATTTATAATAATTAAAAATATGATTATAATTGTTATAATAATTTTTATTTAAATTATTTGATATTATATATCTAGTATTTAAATATAAACTATTTAACCCATATATTCCTTCAAATATTTGACCTATATTTATTCTAGAAGGTATACTTATAGCACTTATAAATATATCAGGTTGAATTTTATTATTTAAATATGGTATATCATTTATTTCACTAATATAAGATATAATACCTTTATGTCCATGTCTATTACATATTTTATCTCCTAATTGTAAATATTTTTGTATACCAATATATATTCTAAATTTTAAATATATATTATTTTTTTCTGTTTTATTATATAAATGATTTGGTAATATTTCTATTTTAATAACCCTACCTATATCATGAATAGTAGAAATAATAGGTTTATTTTTAAATATTCTTAATTTACTACCAAATAAAAAATTAATAATATTAATTAAACTTTTATTATTAAATATAAAAGGCATAAACATTAATTTTGAAATTAATATATTATTAGCTAATATATATGTACCTTCTTTTATTATACCATATTTATCTAAATGTTTAATATTTTTATAATACATTTTAGATAAATTTATACTACATATTTCTGGTATATTATTAATTATATTTAAAGATATTTCATAAATATTCAAATGTAATGAAGTATATAAATTATTATATAAAATTTTTCTACTAATTATAATAGCATCTTCATATTCATATCCTAAATAAGAACCATAACCTACTAATAAATTATTTCCTAAACTATATTCACTATTTAATAAATTAGAATTTATAGCTAAAATCTGACCAATATTAACTTTTTCCCCTACCCATACAATAGGTTTATAAATTAATAATATATTTTGATTTATTTTTTTATAATTATTTAAATAATAAATTATTTGTCTATTAAATAAATCTCTTATTATTATTTTTATACAAGAAACATATATAACTATTCCTTCTTGATATGAAATAATTAAATGATTTAAATATTTATTTAAAATAAAATTATAATTAGTAATAATATTACTTAAATTTGGATATATAATAGGAACAATTTGAGTATGCATTTTTATACTCATTAAATTTCTAATAGAATCATTATAATGTATAAATGGTATTAAATTTTCAATAAAAGATAATAAATAATTAAAAGGTATGTAAATAATATTTTGTGTTATATTACATATTTTAAATGTATTTTTATTTATTGTTAAAATAGTTGTTTTATTAAAATTAATATTTTTTTTTAAATAAATATTATTAAAACTAATATTATAAAAATTTTTATTAAATATATTTAATAATAACTTAAAATTATATCTATTATAAAATATATGTTTATAATATATTACGAATAAATATTTTAAATTTAAAAATATATTTGTAGTTAAATAATTAACTAAACCACAAGTTAATCCTTCATTAGTATTTATTAAACTGATATACCCTAATATATTTCTAGGTAATTCTCTTAAATCATTATTTAAAATAAATTTAGAATTTAATCCTGTAGTTATCATATTTATTTTAAATTTTTGATTTATTTCAGATAAATTATTTACTTGATCAGAATATTGAACTAATGGATTTATATTTATATTTTCTAAAATTATATTTATATATTTTTTATTATTTAATAATAAAGTTATATTATTATATATATTTCTATTAATATTTAATAATTGATATTTAAATATTTTTAAATATTTTTTACTTTTTATTAATAAATTATCAATAATAGAATAAAATTTTTTATTATAAATATTATTAATATAAAAATCAGAATAATATGAAAAATTTAATTTTATTGAAAATAAAATATTTAATAAATTTATATATATATTATTAAATTTATTTAATTTAATAATAAATAATTTTAATGATATTATATTATTAATATTATTATATTTTGAATAAATAAATTTTATATAATTATATATTAATATCTTTTTATTTATAATATTATTATATATAAATAATGATATATTTTTATTTATATATATATTATTTAAATATAATAATAAAATTAAAAAATTAAATTTAAAATTATTAAAATAACAATCAATATTTAACTTACTTATTTTAAAAATAATTCTTAATCCTAAACTAATATATATATATACATATATAATATTTTTATTATTATTTTTAAATTTTATAATAAAAATTTTATTATTTTTTTTAAATAATTGAATACATGTTTTATATAATCCATTTAATATAATAATATTATTATAAATAAAAGGTAATATAAATATTAATATATTAAATTTTATAATTTTATTTATATTTATAAAATTTAACTTTAATGTTAAAATTATTTTTAATAAATTATTTATATTCTGAATAGTATCAATAGAATTTATATTTATATTAGTTAATAAAATTATAATTTTATAATAAATAAAATTAAATTTAACATTTATATTATTATTTAAAAATAAAATATAATATCTTAAATTATATATTATTTCCTGAATTAATAATAAATATAAATTTGATATTATATAATTATTTTTTACTAAAATAGGATTAACAATATATATCATTTAATTTATATTAAATTTACCTATTTTAAAAATATTAATTAAATTAAATGTATTTAAATTAAAATATAAATATATTATATCATTATTATTAAATTTTAATAAAATAATATAATTATTATTTTTATTATTTAATAAAATACCTTTAAAATTAAATATTTGTAATTTATTTTTTATATATTCATAAAAATTTAATTTAATTATTAAATTTTTATTTAAAATAAATTTTTTATTTATTATTATTTTTTTTTGAAAAAAATTTTTATATTTTTTATAATAATATTTTTTAATTATATTAATTTTTATCATATTATTTTTTAAATAATATTTAAATATATTAATTTCTTTAAAATTAATATATATTTTTTATATAATATATAAATAATATTTTTATTTTTTTTATATTTTAATAATTTTTTATATTTATTAATATATAAAAAATTTGTATTAATTACATTTTTATTATATTTCATATTTAATTAATTAAATATATCTTTAATTTTTAATGAAAATAATATAGGTATCTCTAAATTAAATTCAAACGGTAATTTATTATAAATATCAGAACAAAAACCTATAATTAATAATGAAATAGACTCTGAAATACTTAAACCACGTTGCATTAATAAAAATAAATATATAATTTCAATTTTAGAAACAAAAGCTTCTTGTTTTACATAACTAGTATTATTATAATTTTTTATATAAGGAATAGTTACTGTTAAAGAATTATTACCAAATATTAAAGAACTACATTCAGTATAATTATAAGATTTATATGAAAAAGGTTTAATATATACTAAACCTCTAAATATATTTAATGAGTTATTTAAAGATATACTTTTAGAAATTATATAACTTTTAGTATAAGATCCTATATGATACATTTTACTACCAGTATCAGCTATTTGCATATTTGATATAAAAGATATTGAATAAAAATTACTAATAGAAAATTTACCTTTTAAAATAGTAGAAGGGTATTTCCATGTTATAATCGAACCTACTTCAACTTGTATCCAATCTAATTTTGAATAATTTAAACATATACCACGTTTAGTTGTAAAATTATATAAACCACCATTACCTAAATAATCTCCTCTATACCAATTTTGTAATGTATAATATTTTATATAACCATAATCTTTTACTATTATTTCTACTATAGCTACATGTAATTGTGATTCTTTATATAATGAAGCTGTACATCCTTCTAAATATGATACATAAGAATATTTACCAACTATTATTAAAGTACGTTCAAATTGTGCAAAATCAGAAGAATTAGTTTTAAAATATGTTGATAAATTAAAATTACACTTTACATATTTAGGTATATAACAAAAAGATCCTTCACTAAATATTATTGAATTAATATTAGCAAAAAAATTATCTTTATAAGAAATAATAGTACCTAAATATTTTTTTATTAATAAAGGATATTTAAATATAATATCAAATAAAGGTAAAAAAATTATTCCTAATTTTTTTAAAAAATATTGTGTAGTATGTAAAATAGACATACTATCAAATATAATATCTATAGAATTATTTTTTATTAATATACTATCTAAAAATTCAATATTTAAATTATTTTTTAAATAATATATTAAATTATTATCTTTTAAAATAGAAGAATAATAAATAATATTATCATAATTTATATTTGGACAATCAAAAAAATTCCAATCAGGTAATTTAAATATATTTAATAATTTTAAAGAATATTTTTTAAAATTATAAATAAACATATATAAAAAAATATTACTAGATAAATTTTTTATTAAATTTATATTTAATCCTTGTCTTATTAAATATAAATTTATTTTATTTTTATATTGATATTTATAATTTAAATTATAAATATTTAAAAAATTTTTTAATTTTATCATAATAATTATATATAAAAATAATAAAAGCTAATGGTGAGATTTGAACTCATAATCTACTGATTACAAATCAGTTGCTTTACCAATTAAGCTACTTTAGCAAATATAACAATAAATAATTAAATATTCAACTTATTAGGAATTATACACTAAATATATTACTATAAATACATACTAATCCTATAAAATAATTTTTCTAATTATTGTTTTATTCATTTATATGATTAGAATATTATTTTTAACTAAATTTTCTTATTTATATTACTTCAACAATTAAAATTTTATACTTAACTACTCAACTTTACAAAAAAATTTTATAATTGATATATCATTGGTATAATTTTTTTGATCCTCTCGTACTAAAAAAAATAATTTCAATATTCTAACACTTATATTAGATATGGACCGAACTGTCTCACGACGTTCTGAACCCAGCTCACGTATCGCTTTAATAGGCGAACAGACTTACCCTTAAAACATACTACTGCTTTAGGATGCGATAAGCCGACATCGAGGTGCCAAACCTTTTCGTCAATATGGACTCTCGGAAAAGATTAGCCTGTTATCCCTAGAGTAACTTTTATCCGTTAAGCGATAATTTTATTACTAAATAATTATCGGATCATTAAGACCGACATTTATCTCTGTTTAATTTGTAAATTTTACAGTTAATTATATATTATATCTTTATATAATAAATATAACATTGTACTCCTCCGTTTATATATAGGAGGAGACCGCCCCAGTCAAACTATCTCATAAATATTGTTTAAAAATTTGTTATAAAATTTCTATAAGAATTTATATATAAATAAAATGGTATTTCATTTTTAACTAAATTATTTCCAAGAAAATAATATTATTGTTTCCCATTTATACTATGTTAAATATATATATTTTCATTATTTATTAATAGTAAAGCTTCATAGGGTCTTTCTGTCCTAATATAAGAAATCTGTATCTTCACAGATAATTTTATTTCATTAAGATTTTTTTTAAGACAGCATTTAAGTCGTTACATCTTTCATGCAGGTCGGAACTTACCCGACAAGGAATTTCGCTACCTTTGGACCGTTATAGATACAGCCGCCGTTTACTATAGCTTATATATATATTATAATTTAATTACATATATTATTTTAACATAATAGCACTGGGCAGATGTCAATCTTTATACATCATTTTTCAATTTAGCAAAGATTTGTGTTTTTGTTAAACAGTCGCTTAAATTTTTTATTTTCAACTAAAAAGTATCTTTTATCCCTAAGTTACAAGATTAAATTGCCGAGTTCCTTAAAAAAAATTATCTTAACTTCTTAATAATATATATATATTTACTAGTGTCAGTTTACGGTACGAATATATTATAATAAATATATTAATAATTTTTATATAATATAAATAATATTAAAATTATTAATATTAGTCTTAAAATATAAATTATAATATAGTATAAGAATATTAACTTATTATCTATCAATTACACGTTTCATCTCATTTTAAGATTCGACTAACCCTATTAAAAAAAATTATAAATAGGAAACCTTAAATTATAGAAGTATTGGATTTTTACCAATATTTACATTACTCAAATTAGCATTATCACTTTTGATTTTATTATTTTAATTTACATATAAATAATATTTTATCAAAACGCTCTTTTACCAATTTAATTGTACTAATATTAAATTTTATAATATCGATAATTAATTTATTTTCGATTATTTTTGAACTAAAATTATTAAATTAATGAGCTTTTACGCACTCTTTAAAAGATAACTGCTTCTAAATTTACTTTTTAATTATTAATATAATTTTATATTCTTTTTAAAACTTAATTAATATTTAAAAATCTTAATTTATAATTAGGGCTGTTTCCCTTTTGAAAATAAAGCTTATCCTTTATTTTCTAATAATATATATATTTTATTAAATAAAATTATTAAATTATTAATATTAATATTAATTATTTAAATTAATCTAATAAAAAAAGAGTTTTACATTTATTTATATATAAATACTATACTTACATATATTTCAAAGAGAACCAGCTATCTTCAAATTCGATTGGCATTTCACCACTAATTATATTTTATTTGATACTTTTGCAACAGTAATCAATTCAAACTATAATTTAATTTTATTTAAATTTTATTTTAAATATAATTAGATCATTTGATTTCGGGTCTATAATAAATAATATATTTTAAATTTATTAAAAAAATAAATTCGATTTAACTTTGGCTTCATTATTTAAATATATTTAACCTAATTATTATACTATTTATTATAACTTGCTAATTCTTTCTTCAACAAGAATATAATAAAATTAAAATTAAATTTTATTATAATTTATTTAAATTAAAATTCAGATTCTTTTCACTATTTTTTCAAAATTCTTTTCATCTTTCCTCACGGTACTTTTCTCTATAAACTTTTATTATATTTAATTTTATAAGGTAATTCTTATTATTTTTTATATTATTTATATAAAATTATATATTATATTACTTTATTAAAATTTTACATATTTTTTTAAATGTTTATTTTTTTTCAATTCGCTCGCCGCTACTATGAAAATCGTTATTACTTTTTATTCCTTTAAGTACTAAGATGATTCAATTCCTTAAGTTTTTAAATAAAATATTTATATAAAAATATTTTATCAGATACTTTTATAATAATATTTATTAATAAATATAAATATATTTTTATTAATTATTATAAAAATTTCGTTAATATATATAACGTCTTTCTTATTTAATAAAAATTATAGACATCCTTTTAAATTTATTATATATATTTAATTATATATTTAATTTAAATTATAATAAATAAATTTATATAATTATAAGCGAAAAACGGAATTGAACCGATTACCTTCGGAGCATGAATCCGACGAACTTTCCTTATGCTCTATTTCGCTAAAAATAAACTTGAAAAGAATTGAACTTTTATTTTATAATTCGTATTTATATATTTTATCCATTAAATTACAAGTTTATTTATAAATATAAAATTTAAAGTAATTAACTTAGAGGTAAAGTTTCTGCTTTACATACAGAAGACCATTGGTTCGAATCCAATATTACTTAAAAATCTATAATTTAATGGATAAAATAAAAACCTTCTAAGTTTTATATGTAAGTTCAAATCTTACTAGATTTATATATATATGAATATGGCGAAATAGGTAAACGCACTAAATTTAGATTTTAGTTATTATAATAAGAGTTCAAATCTCTTTATTCATATTTATTTAAACTTCTTAAACTAGAATTGAACTAGTGTATTTCGGTTAACAGCCGAATGCTTTAACCACTAAGCTATTAAGAATATTTATATTTATATATAAATATATATTTGGGAATATAGTTTAATGGTAAAATCTTATTTTTGCATAATAAAGATAGTAGTTCAATTCTACTTATTTCCATATAATTTCTATATATGTTATTTATATATTTAAAATATATATTTTATTATATTGCGAGTTTGATCCTAGCTCAGAATTAACGCTAGAAATATACATTACACATGCAAATTAATGATAATATCATAGTGTATAGGTGAGGATATATAAATTTTTAATTTTAAATAGATTATAAATTTTATTAAATAATAATCTATAAGCGCAAAAATATATGTACTATATTAAAAATTAATATTATTTAAAATAAAATTTATATTTGATTAACTAGTTGGTAATTTAAAAGACTACCAAGGTTATTATCAAAAATTGGTTTGAAAGAATGTACAATCACATTAGGATTGAAATAAAGCCTAAATTTTTATAAAAAATCAGCAGTGAGGAATATTTTACAATAAGTGTAAGCTTGATAAAGTAATATTTCTTTTAGGAAGACAGTATTATTAAAATATTGTAAACTTTTTATTTTATTTTTAAATATTGATAAAAATAAAAAATAGTATTTGCTATTTCTGTGCCAGCAGCAGCGGTAATACAGAAAATGCAAGCGTTATTCATTTTATTAGGCGTAAAGCGTTTTAAGGTTTTATATTAATTTTATGTTTAAATATTTAAATTAAATTTAAAATAAATTAATAAATAATAATATAATAGAGTATTATAAAAGTATTAAGAATTTTTTGAGAAGTAGTGAAATACAATGATACAAAAAAGAATATCAAAGGCGGAAGCATAATACTATATAATTACTGACACTTAAAAACGAAAGCTAAGGTAGCAAATAGGATTAGATACCCTAGTAGTCTTAGCTGTAAACTATGAATATTTTATATTTATATTTTATAAATATAATAACTAACGTGATAAATATTCCGCCTGAGTAGTATATTCGCAAGAATGAAATTCAAAGGAATTGACGGGAGCTTATACAAGTGGTGGAACATGTGGCTTAATTCGATGCAACACGATAAACCTTACCAAAATTTAACAATATTTTTAATATTAAGAAATTAATATTTTAATAAAATATATAGGTAGTGCATGGCTGTCGTCAGTTCGTGCTGTGAAGTGTTAATTTTAGTATTATAACGAACGTAACCTTTTATAAAAAAAATTTTTATAATAAATAATAATAAAGATTACGTCAAGTCATTATGCTCCTTATATTTTGGGCTGCTCACGTGTTACATAAAATATTACAATATTTTATTATATGTTAAATATAATAATTAAAATATATTTATAGTTCAGATTATAAATTGAAACTCATTTATATAAAGATGGAATCACTAGTAATCGCTAATCAGAATTATAGCGGTGAATAAGTTCTTAAGCTTTGTACACACCGCCCGTCACATCTAAAAAGTATCATATTATATAAAAATTATTGTTAAATAATAATATATAATTATATAATTTAGATGAAGTCGTAACAAGGTAGCCGTACTGGAAGGTGCGGCTGGATAATAAAATAAATTTTTGGTTGATTTATTTACATAATAAATAAAATAATATTTATATATAAAACTAATAAATTTATTTATATATAATTAACAATTTTATAGACAAAAATAATATTAATACACATTAATGTAAATTTAGTTAAATATTATTTTAATAATTTATAGGTTTTTAGTTTAATGGTTAAAACATACTCTTGATAAGGGTAAAATTTTAGTTCAATTCTAAAAAAACCTATAAAAAAAATAGGATC

**Appendix 2.** Target sequences for *Plasmodium falciparum* 3D7 Primers, Geotyping SNP loci.

>Pf_MT | Plasmodium falciparum 3D7 | 2650 bp ( n:1-2650 )

Features: Geotyping sites mt772, mt853, mt973, mt1283, mt2383

AAGCTTTTGGTATCTCGTAATGTAGAACAATATTGAGTTGACCGTCAAATCCTTTTCATTAAAAGAGTGGATTAAATGCCCAGCCAACACCATCCAATTTGATTGGGAATTATCTGTGTTACAAATTTTTGATCCCAGGCTGGTAAAAAATGTAAACTTTTAGCCCATAAGAATAGAAACAGATGCCAGGCCAATAACTCAAACAGAGCTATGACGCTATCAATTTTTAGCAAGACGGATAAATTTTTCATAGAACTTAACGTATCATCATCCATGCAAAGATAAAACGGTAGATAGGGAACAAACTGCCTCAAGACGTTCTTAACCCAGCTCACGCATCGCTTCTAACGGTGAACTCTCATTCCAATGGAACCTTGTTCAAGTTCAAATAGATTGGTAAGGTATAGTGTTTACTATCAAATGAAACAATGTGTTCCACCGCTAGTGTTTGCTTCTAACATTCCACTTGCTTATAACTGTATGGACGTAACCTCCAGGCAAAGAAAATGACCGGTCAAAACGGAATCAATTAACTATGGATAGCTGATACTATCAATTTATCATTACTCAAGTCAGCATAGTATATATGAAGGTTTCTATGGAAACACACTTCCCTTCTCGCCATTTGATAGCGGTTAACCTTTCCTTTTCCTTACGTACTCTAGCTATGAACACAATTGTCTATTCGTACAATTATTCATATATATATTTGAAACAGGACATACATGTTCATTTATTCTGAATAGAATAAGAACTCTATAAATAACCAGACTATTTCAACAAAATGCCAATATAAAATTGTAATTTGATCAGTGTGAGGTATAACAATATATGATATACCGAAAGAATTTATAAACCATTCGGTAGAAGTATCATATATTTCTATTATTCTTATAAAGTATATTATTAATAATAATAAACCTATTACTACATGAGAAAAATGTAATCCTGTAACACAATAAAATAATGTAGTATATACAGTATCATTTATATGATATGATAAATGTAAATACTCTGTAGTTTGTAGAGATGCAAAACATTCTCCTAATAAGTATATTATACAAATAATACTAGAGATTTCAAAACTCATTCCTTTTTCTATAAATACTTGTAAACATGCAGTCATACATGATGCACTAGCTAATATAAATGTAATTGTTAAGATTAACATTCTTGATGAAGTAATGATAATACCTTCATTACTTAATGGATATGGTGATAAACTAAAATGTAATATACCCCAAAAATATGTAAAGAATAATAAAGCTTCTGATATTATGATAGATAACATACCAGAAGTTAAAGATGAAAATACAGAATAAAAACTTTCTCGAATAGAATATACAAATATTAATAGGATTATAGGGTTAAATGTAAATAATATCCCTACAGAAAAGTATTTTAAAGATGTACCATATAATGATGTTAATGCAGGATATGAAACTAGATGTGCTTTTATATTTGATAAATTACTAAATAAAATAAATTTATAAGAACGGTGAGATAATGTGCCGTAAACATATAACGGTAAGAAGGTTCGCCGGGGATAACAGGTTATAGTATATATAGAGCTCTAATCTTTATATACTATTGGCACCTCCATGTCGTCTCATCGCAGCCTTGCAATAAATAATATCTAGCGTGTATTGTTGCCTTGTACACACCGCTCGTCACGCAATATCAATATACTGGGTATAGAACTCCAGGCGTTAACCTGTAGAGTTGAGATGGAAACAGCCGGAAAGGTAATTTTACGCCCTTAACGTAAAGATCATTTATGAAATAGATTAGCATGGGACTAAAAAATGTTATGTTGTTGGTTTAAGCCCTATTACCATACAAGAGATCGCGTACTTTGGACCGAATAAAGCTGTGAGGAAACTACATTAAAGGAACTCGACTGGCCTACACTATAAGAACGAACGCTTTTAACGCCTGACATGGATGGATAATACTCGACTCTTCCAAAGTATAACCGCTGTCGCTGGGACTGTATGGATCAAATATTTCTCATTTATATCCGAGCCTCATGTTATTTTTATTGTTTTAAATAGATATTCACTTATTACAAATTGTAACCATAAAACTTTAGGATTATACTATTTATGGTTTTCATTTTTATTTGGTAGTTATGGATTTTTATTATCAGTAATACTACGTACTGAATTATATTCTTCATCTTTAAGAATAATTGCACAAGAAAATGTAAATCTATATAATATGATATTTACAATTCACGGAATAATTATGATTTTTTTCAATATAATGCCAGGATTATTCGGAGGATTTGGTAATTACTTTCTACCTATTTTATGTGGATCTCCAGAATTAGCATATCCTAGAATTAATAGTATATCTTTACTGTTACAACCAATTGCTTTTGTTTTAGTTATATTATCTACTGCAGCAGAATTTGGTGGTGGAACTGGATGGACTTTATATCCACCATTAAGTACATCTTTAATGTCATTATCTCCTGTAGCTGTAGATGTAATAATTTTTGGTTTATTAGTATCTGGAGTCGCTAGTATTATGTCTTCATTAAATTTTATTACTACAGTAATGCATTTAAGAGCAAAAGGATTAACACTTGGTATATTAAGTGTTTCTACATGGTCATTGATCATTACATCAGGAATGTTATTGCTAA

>Pf_Api 1 | Plasmodium falciparum 3D7 | 856 bp ( n: 14392-15247 )

Features: Geotyping sites apico15131

TTAGTTAATAATCCAGAAACCCATTTATTTATATATAAATTATTTGTTAAATTACATATTTTAATTGTAAAATTTTTTATTAAATTATTATTATTAATAAATAAAATTTTATTATTCATTAATGATATATTATAAATATATAAATATAACTTATATAAATATAAAGCTATAAATGTAAAATTTAAAATACAATAATTAAATTTTATTTTATATATATATTTATAATTATCAAAATAAATATTCTTATAAATATTTCCTATATAAATTTTTGATTTTAATAAATTATCAAAAGTAATAAACATATTTTAAAAAAATTAATTTACTAAATATCTATACCATCCATTACCAACAGGTAATAAATCAGTCAATATTATTTTAGATTTTATATCTATTAACCAATCAATTTTATTATTTAAAATATTTAAACTTATTATTTTAAATGTATTCTGAAAACTTATATTTGTTAAAAATCCAGAATTAGCTAAAATTGATTTTGTAATACCTAAAATAATTGGTTCATATTTATAAATATAATGTTTATTTAAATTTAAAGAATAATTAATTATATTTATTAATTGTAAAGATATTATATCATTATATTTAAATATTTTAAAATTATTAGATATTATCTTTATACATGATAACATTTTTTTTATAATAAGTTCAAAATAAATAGAAGGTAAATAAATATTTTGATAACTATATTGTTTCAAAATAGATTCAATTAAAATATTATATACATATATATATGAACTTTTAGTTGCTTGATAAATATTTATAGATTTTAATAAAAATCTAAAATAATATTTTAAATTTTTATTAATTGAATAAAATTCTGTATGTAAAGAATATCCAC

>Pf_Api 2| Plasmodium falciparum 3D7 | 806 bp ( n: 1473-2278 )

Features: Geotyping sites apico2122

TGCCTGAGTGGTTAAAAGGAATGGACTGTAAATCCATTGATAATATATCTACATCAGTTCAAATCTGATTTAACTTATTTTTATATATAAAGAGAAATGACTGAGAGGTTTATAGTTATAAATTGCTAATTTATTGTATATATAATAATATTATACCAAGGGTTCGAATCCCTTTTTCTCTATTATTAGAATTTGTTAGTTTAATTAGGTAAAAATATTATTTTGTCATAATAAAGAATACGAGTTCAATTCTCGTCAAATTCGTTTTATTTAAGAATTACTAGCTTAATTGGTAGAGTACTCGACTTTTAATCGAATGGTTCTGAGTTCAAATCTCAGGTAGTTCATTTAATAACTTTTATCGTTTAAAGGTAAGACATCTTTTTTTCAAGAAGAAAATAGGAATTCGATTTTCCTTAAAAGTATTTATTTAATTCAGAATATAGTGTAATGGTAACATATCTATTTTGGGAATAGAAGAATATAGGTTCAAATCCTATTTTTCTGAAAATAATATTATATGTATATTTTAGAATGAATATTATTATTTTAAATAATAATACATTAAATAATATAATATTTAAATATAAATATAATTTTTTTATTAAATTATATTTTAATAATTATATTAAAATATGTAAATTAATAATTTATATTATAAAATATTTATATATATATAATATTTATATGTATAAACATACTAAGAATAAAAGTAAAGTATATTTTAGTAATAAAAAAATAAGAGTACAAAAAGGGCTAGGAAAAGCTAGATTAAAAAATTTTAAATCACCTGTATGTAAACAGGG

>Pf_Api 3| Plasmodium falciparum 3D7 | 1679 bp ( n: 19599-21277 )

Features: Geotyping sites apico20831, apico21188

TCTTTGACCCCCTTGTTTTGTATTACCTTTTATTGGTTGTTGAGTTAATTCAGAATATAAACCTATAAATCTATATCTAAATTTATCTTTTATCATATGAATTAATTTATAATAATAAATATTATTTAAACAAATACTATTATTAATCATATTACCAGTAAATGGATTTTTTAAATAATATTTATTATAATTATAAGACATTTTACTATTTATATTAAAATTATTATTATAATTATATTTATAATAATTAAAAATATGATTATAATTGTTATAATAATTTTTATTTAAATTATTTGATATTATATATCTAGTATTTAAATATAAACTATTTAACCCATATATTCCTTCAAATATTTGACCTATATTTATTCTAGAAGGTATACTTATAGCACTTATAAATATATCAGGTTGAATTTTATTATTTAAATATGGTATATCATTTATTTCACTAATATAAGATATAATACCTTTATGTCCATGTCTATTACATATTTTATCTCCTAATTGTAAATATTTTTGTATACCAATATATATTCTAAATTTTAAATATATATTATTTTTTTCTGTTTTATTATATAAATGATTTGGTAATATTTCTATTTTAATAACCCTACCTATATCATGAATAGTAGAAATAATAGGTTTATTTTTAAATATTCTTAATTTACTACCAAATAAAAAATTAATAATATTAATTAAACTTTTATTATTAAATATAAAAGGCATAAACATTAATTTTGAAATTAATATATTATTAGCTAATATATATGTACCTTCTTTTATTATACCATATTTATCTAAATGTTTAATATTTTTATAATACATTTTAGATAAATTTATACTACATATTTCTGGTATATTATTAATTATATTTAAAGATATTTCATAAATATTCAAATGTAATGAAGTATATAAATTATTATATAAAATTTTTCTACTAATTATAATAGCATCTTCATATTCATATCCTAAATAAGAACCATAACCTACTAATAAATTATTTCCTAAACTATATTCACTATTTAATAAATTAGAATTTATAGCTAAAATCTGACCAATATTAACTTTTTCCCCTACCCATACAATAGGTTTATAAATTAATAATATATTTTGATTTATTTTTTTATAATTATTTAAATAATAAATTATTTGTCTATTAAATAAATCTCTTATTATTATTTTTATACAAGAAACATATATAACTATTCCTTCTTGATATGAAATAATTAAATGATTTAAATATTTATTTAAAATAAAATTATAATTAGTAATAATATTACTTAAATTTGGATATATAATAGGAACAATTTGAGTATGCATTTTTATACTCATTAAATTTCTAATAGAATCATTATAATGTATAAATGGTATTAAATTTTCAATAAAAGATAATAAATAATTAAAAGGTATGTAAATAATATTTTGTGTTATATTACATATTTTAAATGTATTTTTATTTATTGTTAAAATAGTTGTTTTATTAAAATTAATATTTTTTTTTAAATAAATATTATTAAAACTAATATTATAAAAATTTTTATTAAATATATTTAATAATAACTTAAAATTATATCTATTATAAAATATATGTTTATAATATATTACGAATAAATATTTTAAATTTAAAAATATATTTGTAGTTAAATAATTAACTAAACCACAAGTTAATCCTTCATTAG

>Pf_Api 4| Plasmodium falciparum 3D7 | 510 bp ( n: 23595-24104 )

Features: Geotyping sites apico23803

GAAGCTGTACATCCTTCTAAATATGATACATAAGAATATTTACCAACTATTATTAAAGTACGTTCAAATTGTGCAAAATCAGAAGAATTAGTTTTAAAATATGTTGATAAATTAAAATTACACTTTACATATTTAGGTATATAACAAAAAGATCCTTCACTAAATATTATTGAATTAATATTAGCAAAAAAATTATCTTTATAAGAAATAATAGTACCTAAATATTTTTTTATTAATAAAGGATATTTAAATATAATATCAAATAAAGGTAAAAAAATTATTCCTAATTTTTTTAAAAAATATTGTGTAGTATGTAAAATAGACATACTATCAAATATAATATCTATAGAATTATTTTTTATTAATATACTATCTAAAAATTCAATATTTAAATTATTTTTTAAATAATATATTAAATTATTATCTTTTAAAATAGAAGAATAATAAATAATATTATCATAATTTATATTTGGACAATCAAAAAAATTCCAATCAGGTAATTTAAATATA

>Pf_Api 5| Plasmodium falciparum 3D7 | 1401 bp ( n: 3857-5257 )

Features: Geotyping sites apico4370, apico4878, apico4945, apico5005

AAGTACATCCTTTAATATTTAGAGGATTAATATATAAAAACTATTTAAATAATTTTTATATAAATATTAGTAAAAATAAATATTATTTAATTAATATATTATTAACATATTTTATTTATTATAATATGTATAGAATATGTAATTATAAAGAAGATAATTATATTAATATAAATATAAATTTTAGTATAAATAAATTTGTAATTACATTTTTTTTATATAATAAATATTATAATATATATAATTTAAAATATATATTTATTTTATTAAATTATTTTAATTATTATTATTATAATTATTATAATTATATGTGTTTTTTAAAAATAAAATATGTAAATAATATAAATAATATTAATGTTATAATGTATTATATAAAAAAATATTATATAAAATATAAATCTTTAAAATTAATATTTGATTATTTATATAATAATATTTTAAAAAAAAATAATTTTAATATAAAAGGATTAAAAATTAAATTTTCAGGTCGTTTTAAAAATAGTTTAAAGACTAAAATAGAAATATATATTTATGGTATTATATCTTTAAGTACATTAAATAATAATATTAAATATATAAATGATATTATAAATACTAAATATGGTATTTTAAGTATAAAAATTTGGTTAAATATTTAAAAATAAATTAAAAAAATTTATAATATATGACTAATATTATAATAAAAAAAAATCAAAAAGGTAAAATAAAAGGTAAATTTAATTTAAAATTTTTAAGTTTATATTGGGGTATTATATCTTTAGATTCTGGATTTTTAACAAAAAATCAATTAGAGACTTCAAAATTTATAATAAATAAATATTTAAAAAAAATAGGAGTATATAAAATTTGTATAAGATGTATAAAATCATTAACTAAAAAATCTTTAAAAACTAGAATGGGATCAGGAAAAGGATCTATAGAATTATATGTAAGTCCTATAAAAAAAAATAAGTTATTATTTGAAATAAGTAAAATTTCAAATAATATTATTTATACTATAACAAAAGTTTTATCTTATAAATTACCTTTTAAATTACAATATATTAGAAAATAATTAAATAAAGAATATGAATATAAAAATAGGATATGTTATTAAGAATTTAAATATAAATATTAAGATAGTTTGTATATCTTTTTATAAGTATAATTTTAAATATAAAAAATTATTATTATGTAATCTATATATAAAAATATATGATAATAGAAATGAAATTATTATAAATGATTATATATTATTTAAATATTATAAAAAAAGTAAATATTGTAATAATAAAGTAATAAAAATTTTATGATATATATAAATAGTATATTAGATGTAATAGATAATAGTGGTATATTTAAATTTAAATATATTTGTACTTTAAATAAATATAAAAATCCTAAATATGGTGATATAGTTATAGG

>Pf_Api 6| Plasmodium falciparum 3D7 | 1640 bp ( n: 5415-7054 )

Features: Geotyping sites apico5715, apico6361, apico6832

GTAGGTACTAAAAGTAATAATTATATATCTAAATATATAAAATATAAATTAAATATGAATAAGTTTAAATAAATTTATTTGAATTATGATTATTAAATTTTTAAATAATGTTAAATATAATTTTAAATTAAAAAAAAATTTTATATTATATAAATATAATAAAGTAATATATTATTTAAGTATATTATTATATAATTATAAATATATATTTAAATTATATATTTTAAATATATATAATAAATATTTTATTTTTATAATTTTAAATGAATATAAAAATATTAATTATTTTAAAGTATATATCAAATATAATCAATTATTTTATATGAATTTTAATAAATTATTATGTTTTATAAAATTTAAAAAATATTTTAAAGGTTTATTAATTTTATATTCTTCAAAATATAAATTTATTACACATATATTAGCTTTAAAATATAAAATTGGTGGTATTTTATTATTTTATATTTTGTAAATATTATAGTTATAATTGTTATGATAAATAATAGAAAGTACATATTTTTAAATAATAATATAAAAGAAGGTAATATATATTTAATTTTAGATATAAAATATTTGAATTATATATATATATTTAAAAATAATAATAAGATATATAATATTAATATATTAAATAATATATATATATATTTTATAAAAAATAATATTTATTATTTTATATTTAAAATGTATAAGTATATATTTAGTAATATATTTAATAAAATACAATATAAAAAGTATCAGTTAATTTTAAATATAATTGGTATAAATTATAAATTTTATTATATAAAAAAAGGTAATTTTTTAATATTTCAATTAAAATATAGTCATAAAATAATTATTAAGTTACCTAATATAATATTTTGTGAGTTAGATATAAATAAAAATTTTATATATTTATATAGTATGGATATATTTATATTAAATTCTATTGGGAATTTAATAAATTCATTTCAATATATAAATAAATATAAAGAATTAGGTATAAAAAAATTAATATGATAATTAATATTATTAATAAAAAATTAAATAATTTTTATATATTATATTATGTTATAGTATTATTTAAAAGTATTTTTATATTAATTTTAAAAAGAAAGTTTAATAATTATTTGTATAAAATTTATAATATTTTAATTATATATTTAAAATTATATTATATTATAAATAATAAGAGAGATAAAAAATATATTTTTAATAATAAAAATGTAAATTTTATTTATTTTTATATTTATAAATATAATAATAATATTAAGTTAAATAATAGTATTATAAAGTTAAATAATAATATTATTGAAAAAATTATTGAAATAAAAAAAATATCTTATACAATAAAAAAAGGAAGAATTAGAAGATATAAAATAGTATTAGTATTAGGTAATAAACAAGGTTGGATAGGATTAGGAGTTAGTAAGAATATCAATATTAATAAAGCTATTATATCTGCTAAAATAAAAGCTTTAAATAATATTTATTATTTTAAATATTCTATATTAAATATATATAAATTAAGATATATATATATTAATTATAGTAAATTTTTTATTAAATTACAATTTAAAATTTATAATTATTTAAATATAAGATTTTTATTATTAAAATATTTATTTGAATGTTTAGGTTATTTTAATTGTAAAATAATA

>Pf_Api 7| Plasmodium falciparum 3D7 | 994 bp ( n: 8785-9778 )

Features: Geotyping sites apico9003, apico9096

CAACATATAAATTTAGGTACTATAGGGCATGTAGATCATGGAAAAACTACATTAACAACAGCTATATCTTATTTATTAAATTTACAAGGATTATCAAAAAAATATAATTATTCAGATATTGATTCAGCTCCAGAAGAAAAAATAAGAGGTATTACAATAAATACAACACATATTGAATATGAAACTTTAACAAAACATTGTGCTCATATAGATTGTCCAGGACATTCCGATTATATTAAAAATATGATTATAGGAGCCACACAAATGGATATAGCAATTTTAGTAATATCTATAATAGATGGTATAATGCCTCAAACTTATGAACATTTATTATTAATAAAACAAATAGGTATAAAAAATATAATTATTTTTTTAAATAAAGAAGATTTATGTGATGATGTTGAATTAATAGATTTTATAAAATTAGAAGTAAATGAATTATTAATTAAATATAATTTTGATTTAAATTATATACATATATTAACTGGTTCAGCATTAAATGTAATAAATATAATTCAAAAAAATAAGGATTATGAATTAATAAAATCTAATATTTGGATACAAAAATTAAATAATTTAATTCAAATAATTGATAATATTATAATACCTACTAGAAAAATTAATGATTACTTTTTAATGTCAATAGAAGATGTATTTTCTATAACAGGTAGAGGTACAGTAGTAACAGGTAAGATTGAACAAGGATGTATAAATTTAAATGATGAAATTGAAATTTTAAAATTTGAAAAATCATCTCCTAATTTAACAACAGTTATAGGATTAGAAATGTTTAAAAAACAATTAACACAAGCACAATCCGGAGATAATGTAGGTATTTTATTAAGAAATATTCAAAAAAAAGATATAAAAAGAGGTATGATTTTAGCAACACCTAATAAATTAAAAGTATATAAGTCTTTTATAGCTGAAACATATATTTTAACTAAAGAAGAAGGTGGTCGTCATAAACCTTTTAATATTGGATATAAACCTC

>Pf_Api 8| Plasmodium falciparum 3D7 | 1918 bp ( n: 10497-12414 )

Features: Geotyping sites apico11066, apico11619, apico11671

CCAAAATTTATAATAAAGGTTCGAATCCTTTAGGACATGTATATAATAATATATAAATAAAAAAATGATTATTTCATGTAAATATAAATTTTTAATAAAAAATAATCAAATAAATTTAAATTCAATTTTAAATTTTAAATTTAAAATATATAACATTAATATTAATTTATTAAATAAAAAAATAACAGAATATATAAATAAATATAAATTGAATTTATTTATAATTTATATTTATTCAGACAAAACATTTAAAATTATATATAATTATACAATATATAATTTATATAATAAATATAATAGTAAAGTAAATAAAATATTACTAATATATAAGATATTATTATATAAAAAATTTCAATTATTATTTTATAATATTAATCAATTATTATATATTATAAAAAATAATTTTAAACAAATAAACATAAAAAATAATAAAAATCATGATAATTTTAAATAATCTTTATTGTACAAAAGAATTAATAATAATATTTATTAAATCTGAATATTTAGCATTAAAATATAATAATAATTTTATAATGCCTATTCATTTATTATTAGGATTATTATTAACTGATAATTTATGTACAAAATTTTTAAAAATAAATAAAAAAATAATAAATAATAAAATAATTTTATCTTTATTAAATAAATATAAATATAATAATAAAAATATTATTAATATAAATTTTTCTAATAAAGTTATTAATATATTAATTAAATTAAATAATTTTAATTTTAAAATTAATTCATTTAATTTATTATTATTATTATTAGAAGAAAAAAATAATAATAAAGATATTAATTATTTATTTAAATATTTAAATTTAAATTTTTCTAATTTAAATTTAAATAATTATATAAAAACTAATATTTTTTCAAATAATATTAGAATAAAATTAAAAGAAATATCAGTAAATCTTTTAAATTTAAATTATATTTATAATAATAATTTAAATTTTTATAAACAACAATATATACAGTTATTACAAATTTTAAATTTAAAAATAAAAAAACATATAATACTAGAAGGGGTAAATGATAATATTTTTATATTTTTACAATTATTAATTAATAATATAAAAAATAAAATTATACCAATATATTTAAAATATACAGAAATATGGGTATTAAATGATTTATTAACTTATGATATACAAACTTTAATATATAAAATATTGAATATATCTAAATATTTTACAAATAAATATAAATTAATCTTAATTATAAAAAATATAGAAATATTTAATCTATCAGATAATATTAATAATGATAATAATAAATTATATTATTTATTTTTATTATTAAATAAATTATATGGATATAATATACATATAATAATAGTAACTAATAAAAAAGAATATAATACATATTTTAAATATAATATAATAAAAGATTCTTATTTTTATAAAATAAGAATAAAAGATTTATCAATATTACAAACATTCTTAATAATAAAAAATAATATATATAAATATATTAATTATTATAAAATTAATATTAATAATTATATTATATATGAATTAATAAATTTAAGTAAAAAGTATATAAAACCTTTAATATTACCTACAACTCCATTAATTTTATTAGAAAATTCATGTTCTAATAAATATTTATTAAATAATAAAATATCTTATTCAAATTTTAATTATTTATTTACATATAATAATAATATTATATATAATAATAAAAATAATAATTTAACTATAGAAGATATTAAAAATTCAATATCTAATTACTTAAATATATCTAAAACCATATTATTTAAAGACAATAAATTAACTAAATTAAATTTAACTAAATTAGAAAATTATTTATATAATCATATATATGGTCAAAATCATATTTTTAATAAAATAATACCTTTTATTAAACAAAATTTTATAGGATTAAAAAATAAAAATAAACCTATAGGAAGTTGGAT

>Pf_Api 9| Plasmodium falciparum 3D7 | 837 bp ( n: 26347-27183 )

Features: Geotyping sites apico26659

AATTCGATTGGCATTTCACCACTAATTATATTTTATTTGATACTTTTGCAACAGTAATCAATTCAAACTATAATTTAATTTTATTTAAATTTTATTTTAAATATAATTAGATCATTTGATTTCGGGTCTATAATAAATAATATATTTTAAATTTATTAAAAAAATAAATTCGATTTAACTTTGGCTTCATTATTTAAATATATTTAACCTAATTATTATACTATTTATTATAACTTGCTAATTCTTTCTTCAACAAGAATATAATAAAATTAAAATTAAATTTTATTATAATTTATTTAAATTAAAATTCAGATTCTTTTCACTATTTTTTCAAAATTCTTTTCATCTTTCCTCACGGTACTTTTCTCTATAAACTTTTATTATATTTAATTTTATAAGGTAATTCTTATTATTTTTTATATTATTTATATAAAATTATATATTATATTACTTTATTAAAATTTTACATATTTTTTTAAATGTTTATTTTTTTTCAATTCGCTCGCCGCTACTATGAAAATCGTTATTACTTTTTATTCCTTTAAGTACTAAGATGATTCAATTCCTTAAGTTTTTAAATAAAATATTTATATAAAAATATTTTATCAGATACTTTTATAATAATATTTATTAATAAATATAAATATATTTTTATTAATTATTATAAAAATTTCGTTAATATATATAACGTCTTTCTTATTTAATAAAAATTATAGACATCCTTTTAAATTTATTATATATATTTAATTATATATTTAATTTAAATTATAATAAATAAATTTATATAATTATAAGCGAAAAACGGAATTGAACCGATTACCTTCGGAGCATGAATCC

**Appendix 3. Informative SNPs in *P. falciparum* mitochondrion and apicoplast genomes**

|  |  |  |  |  | |  | **Location information of mutations** | |
| --- | --- | --- | --- | --- | --- | --- | --- | --- |
| **Organelle** | **Position** | **Gene** | **3D7 reference** | | **Alternate** | **S/NS** | **Geographical Origin** | **Country** |
| ***mt*** | **772** | ***cox3*** | **C** | | **T** | **N** | **Mixed** | **Equatorial Guinea** |
| ***apico*** | **2122** | ***rpl 4*** | **T** | | **G** | **S** | **EAF** | **Equatorial Guinea** |
| ***apico*** | **6832** | ***rps 5*** | **C** | | **T** | **N** | **EAF** | **Congo** |
| ***apico*** | **20831** | ***rpob*** | **G** | | **A** | **S** | **EAF** | **Equatorial Guinea** |
| ***apico*** | **21188** | ***rpob*** | **G** | | **A** | **S** | **WAF** | **Equatorial Guinea** |
| ***apico*** | **23803** | ***sufb*** | **T** | | **C** | **N** | **WAF** | **Equatorial Guinea** |
| ***apico*** | **26659** | **RNA** | **A** | | **G** | **-** | **Mixed** | **Equatorial Guinea, Congo, Sierra Leone** |

*S: Synonymous; NS: Nonsynonymous.
